# Clinical efficacy and multi-omics analysis of Si–Ni–San for depression treatment in breast cancer patients: a randomized, double-blind, placebo-controlled, crossover trial

**DOI:** 10.1186/s13020-025-01283-y

**Published:** 2026-01-06

**Authors:** Shicui Hong, Miao Yu, Yifeng Zheng, Shengqi Wang, Juping Zhang, Bo Pan, Honglin Situ, Li Guo, Shaowen Zhong, Ying Chen, Yunxi Li, Lingling Yang, Yan Li, Zhiyu Wang

**Affiliations:** 1 State Key Laboratory of Traditional Chinese Medicine Syndrome, Chinese Medicine Guangdong Laboratory, Guangzhou University of Chinese Medicine, Guangzhou, China; 2 Breast Disease Specialist Hospital of Guangdong Provincial Hospital of Chinese Medicine, The Second Clinical College of Guangzhou University of Chinese Medicine, Guangzhou, China; 3 The Research Center for Integrative Medicine, School of Basic Medical Sciences, Guangzhou University of Chinese Medicine, Guangzhou, China; 4 Guangdong Provincial Key Laboratory of Clinical Research On Traditional Chinese Medicine Syndrome, Guangdong Provincial Academy of Chinese Medical Sciences, Guangdong Provincial Hospital of Chinese Medicine, Guangzhou, China; 5 Guangdong-Hong Kong-Macau Joint Lab on Chinese Medicine and Immune Disease Research, Guangzhou University of Chinese Medicine, Guangzhou, China; 6 Psychological Sleeping Department, Guangdong Provincial Hospital of Chinese Medicine, The Second Clinical College of Guangzhou University of Chinese Medicine, Guangzhou, China; 7 The Second Affiliated Hospital of Guangzhou University of Chinese Medicine, Dade Road 111, Guangzhou, China

**Keywords:** Breast cancer, Depression, Traditional Chinese medicine, Si–Ni–San, Clinical efficacy, Multi-omics analysis

## Abstract

**Background:**

Breast cancer is a leading malignancy and a significant cause of mortality in women. The coexistence of depression has been associated with a more aggressive breast cancer progression. Si–Ni–San (SNS), a Traditional Chinese medicine (TCM) formula, has been traditionally used for the treatment of depression. This clinical trial aimed to evaluate the effectiveness and safety of SNS in relieving depression in breast cancer patients, and explore its molecular mechanism.

**Methods:**

A randomized, double-blind, placebo-controlled crossover trial was conducted in breast cancer patients with mild to moderate depression (MMD). Patients were asked to participate over a four-week SNS treatment and a four-week placebo intervention in randomized order, with a two-week washout period. The primary endpoint was the change of Hamilton Depression Scale-24 (HAMD-24) score. Secondary endpoints were the changes of Functional Assessment of Cancer Therapy—Breast (FACT-B) and syndrome score of Traditional Chinese medicine (TCMSS). Liver function test (LFT) and mental status examination (MSE) were conducted to ensure the safety. Gut microbiota, serum metabolomics, cytokine profiling and T lymphocyte subsets were tested to explore the molecular mechanisms.

**Results:**

A total of 53 patients completed the trial. Compared to placebo intervention, SNS treatment significantly reduced HAMD-24 score (4.46 ± 4.403 (95% CI − 5.69, − 3.24) *vs.* 0.66 ± 4.463 (95% CI − 1.89, 0.57), *P* < 0.001), accompanied by the improved FACT-B scores (7.22 ± 13.77 (95% CI 3.66, 10.77) *vs.* − 0.78 ± 15.32 (95% CI − 4.74, 3.17), *P* = 0.09) and TCMSS scores (− 12.71 ± 12.88 (95% CI − 16.30, − 9.13) *vs.* − 5.60 ± 4.69 (95% CI − 9.42, − 1.79), *P* = 0.01). No obvious risk in LFT, MSE and fewer adverse events were observed. Exploratory multi-omics analyses revealed three key phenomenon including specific reduction in *Lactobacillus* abundance, decreased serum indole levels, and increased CD8^+^ T cell proportions, suggesting their potential involvement in breast cancer-related depression.

**Conclusions:**

SNS is efficacious for relieving depression in breast cancer patients with high safety, and *Lactobacillus*-Indole-CD8^+^ T signaling may be involved in its pharmacological mechanisms. Further research is required to refine study designs and substantiate the speculated mechanism.

*Trial registration*: ChiCTR, ChiCTR2200065009. Registered 25 October 2022.

**Graphical Abstract:**

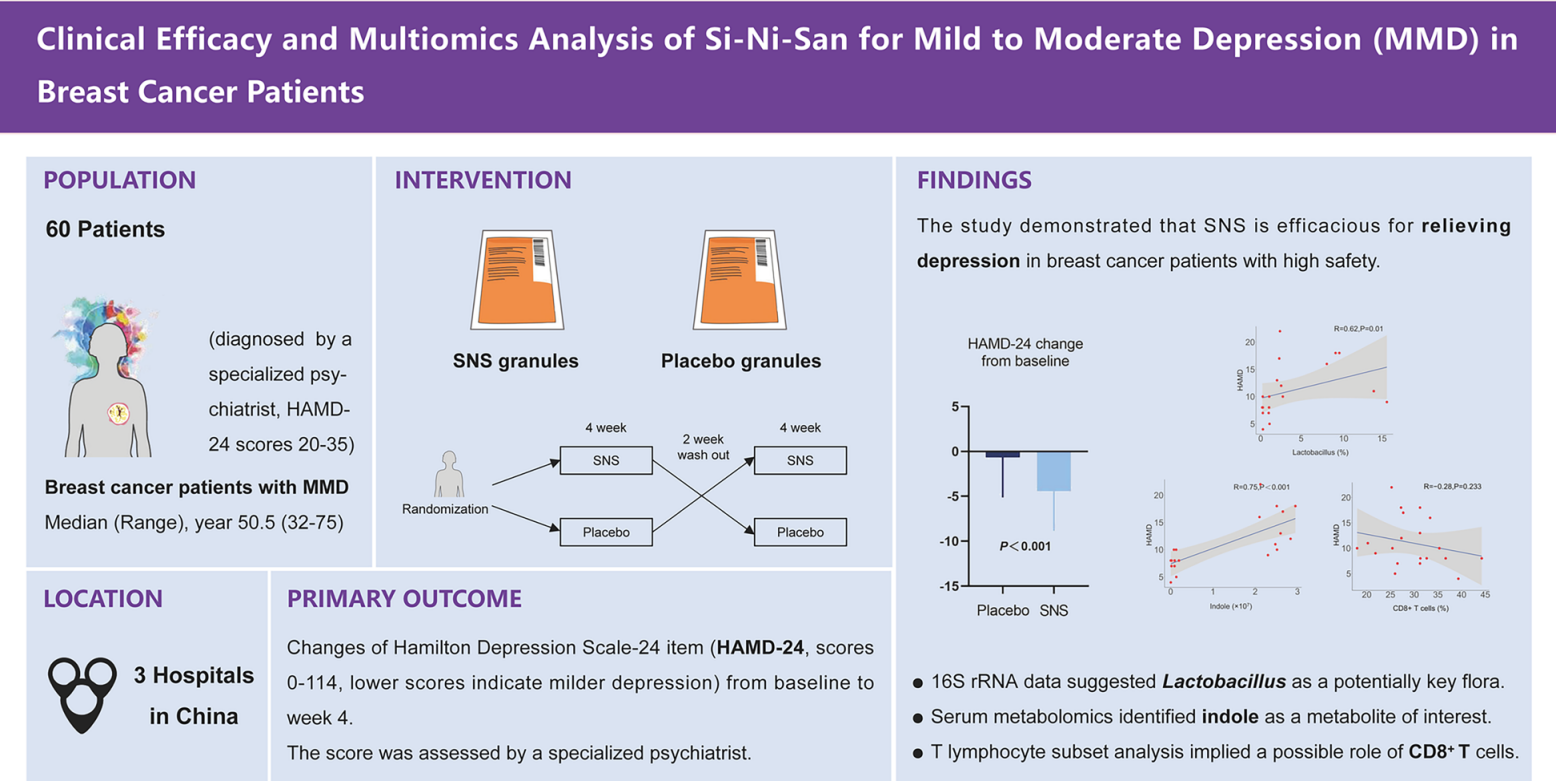

**Supplementary Information:**

The online version contains supplementary material available at 10.1186/s13020-025-01283-y.

## Introduction

Breast cancer has ranked as one of the most common female malignancies, and a leading cause of death among women worldwide. In 2022, there were 2, 308, 897 new cases of breast cancer globally, accompanied by 665, 684 fatalities [1]. It is estimated that by 2040, there will be approximately three million new cases of breast cancer and one million deaths annually, which lead to an overwhelming burden for public health and economic development [2]. Despite advancements in cancer treatment brought survival benefits, patients often suffer reduced quality of life from cancer-related symptoms [3, 4]. In 1998, the Office of Cancer Complementary and Alternative Medicine (OCCAM) was established in the National Cancer Institute (NCI) of the United States of America (USA), with a focus on evidence-based complementary and alternative medicine interventions. It is crucial to develop comprehensive, patient-centered strategies to reduce cancer-related symptoms, improve quality of life, and support the successful reintegration of breast cancer patients into society.

Depression is highly prevalent among breast cancer patients [5]. It has been reported that over 30% of breast cancer patients were diagnosed with depression, which could persist for more than five years after diagnosis [6]. It is noteworthy that comorbid depression can exacerbate breast cancer progression, with a 24% increased risk of recurrence and a 29% increased risk of breast cancer-specific mortality, respectively [7]. In addition, high level of depression in breast cancer patients not only brought great influence on quality of life, but also negatively impacted their compliance to cope with cancer treatments [8]. Diverse biological mechanisms involved in depression-induced cancer progression, including dysregulation of the Hypothalamic–Pituitary–Adrenal (HPA) axis, immunosuppressive tumor microenvironment, imbalanced gut microbiota, and metabolite alterations [9–12]. Ongoing research is focusing on how microbial signals, particularly the co-metabolite signals, interact with the immune system during stress-induced cancer progression [13, 14]. In terms of clinical treatment, specialized psychological and pharmacological interventions are the primary approaches for mild to moderate depression treatment [15]. However, a considerable percentage of patients undergoing chemotherapy or endocrinotherapy face the risk of pharmacological interactions with psychotropic drugs, potentially affecting breast cancer outcomes [16] [17]. Moreover, only a subset of patients benefited from antidepressants [18]. Besides, although timely treatment to cancer-related depression is necessary [19], corresponding clinical application is hindered by the demanding nature of the interventions and high treatment costs [20]. In USA, only one-fourth of cancer patients with depression receive appropriate treatment [21], compared to that of 9.2% in China [22], highlighting the urgent need to develop more feasible and efficacious strategies. In the past decade, the American Cancer Society has endorsed integrative therapies as valuable strategies for managing symptoms during breast cancer treatment [23].

TCM stands out as the most commonly utilized complementary and alternative therapy for both cancer and depressive disorders, especially in China [22, 24]. Recent studies further support the efficacy of Chinese herbal medicine in improving depressive symptoms [25–27], with exploration of pharmacological mechanisms including the hypothalamic–pituitary–adrenal axis, microbiota-gut-brain axis, and other contributing pathways [28–31]. Si–Ni–San (SNS), a classical traditional formula outlined in the “Treatise on Febrile Diseases” (Shanghanlun, 伤寒论) has been appreciated for its antidepressant properties since the Han Dynasty. SNS consists of the four herbs including *Bupleurum chinense* DC. (Chaihu), *Paeonia lactiflora* Pall. (Baishao), *Citrus aurantium* L. (Zhishi), and *Glycyrrhiza uralensis* Fisch. (Gancao) (Table 1). Historically, SNS is widely applied in the treatment of depression-related functional dyspepsia [32, 33], postpartum depression [34], depression following acute cerebral infarction [35], and various other depressive status. Emerging studies have further underscored its potential in suppressing the growth and metastasis of breast cancer triggered by chronic psychological stress [36–38], potentially through interactions with the central nervous system and cancer stem cells [39, 40]. Our prior research has revealed that SNS can inhibit depression-induced breast cancer growth and metastasis by facilitating estradiol metabolism in the liver [41]. Additionally, we found that SNS could suppress chronic psychological stress-induced aerobic glycoloysis of breast cancer [39]. However, despite these promising findings of SNS in improving depression status and breast cancer prognosis, there remains a lack of high-quality clinical trials evaluating the efficacy of SNS in treating mild to moderate depression in breast cancer patients.

**Table 1:** Composition and proportion of SNS

Chinese name	Botanical name	Latin name	English name	Medical part	Weight (g)	Category
Chaihu	*Bupleurum chinense* DC.	*Bupleuri Radix*	Chinese Thorowax Root	Root	10	Chief
Baishao	*Paeonia lactiflora* Pall.	Paeoniae Alba Radix	White Peony Root	Root	10	Deputy
Zhishi	*Citrus aurantium* L.	Aurantii Immaturus Fructus	Immature Orange Fruit	Fruit	10	Assistant
Zhigancao	*Glycyrrhiza uralensis* Fisch.	Glycyrrhizae Praeparata cum Melle Radix et Rhizoma	Prepared Liquorice Root	Tuber	10	Envoy

Here in, a randomized placebo-controlled crossover trial was designed to investigate the efficacy and safety of SNS in alleviating depression symptoms in breast cancer patients with mild to moderate depression (MMD). Besides, multi-omics profiling was applied to explore the underlying molecular mechanisms of SNS linking to the gut microbiota, metabolites, and the immune system. Our findings demonstrated that SNS is efficacious and safe in treating MMD among breast cancer patients, accompanied by the improvement of quality of life and syndrome score of TCM. Multi-omics analysis revealed that the axis of *Lactobacillus*-Indole-CD8^+^ T is a potential signaling for the pharmacological activity of SNS. This study not only presents the first clinical evidence supporting the application of SNS for MMD treatment in breast cancer patients, but also provides a connective network between microbiota, metabolites and immune cells depicting the molecular mechanisms of SNS, which deserves further exploration in the future.

## Methods

### Study design and ethic approval

This study is a randomized, double-blind, placebo-controlled, multi-center crossover trial. Patient recruitment occurred between November 7, 2022 to February 22, 2024 from three branch hospitals of the Guangdong Provincial Hospital of Chinese Medicine (Dade Road Hospital, Ersha Island Hospital, and University Town Hospital). The trial was registered with Chinese Clinical Trial Registry (https://www.chictr.org.cn) under the identifier ChiCTR2200065009. Ethical approval was obtained from the Ethics Committee of Guangdong Provincial hospital of Chinese Medicine (2022-177-01) (central ethics approval covered all sites, Supplementary Material 1.1), and the study was conducted in accordance with the Declaration of Helsinki and Good Clinical Practice guidelines. All participants provided explicit written consent for their involvement in the research and for the dissemination of their data. The study followed the CONSORT guidelines [42] to ensure rigorous methodology and reporting (Supplementary Material 1.2).

### Study population

Eligible patients were breast cancer survivors aged 18–75 years in the rehabilitation phase (≥ 3 months post-completion of surgery, chemotherapy, and/or radiotherapy), receiving stable maintenance medications (e.g., endocrine therapy). They were required to meet the following criteria: a diagnosis of MMD as per the International Classification of Diseases, 10th Edition (ICD-10) confirmed by a study psychiatrist during a clinical assessment; a total Patient Health Questionnaire-9 (PHQ-9) score ranging from 5 to 14; a total Hamilton Depression Scale-24 (HAMD-24) score between 20 and 35; identification of the Liver Qi Depression Pattern (LQDP) based on the expert consensus of the Neurology Committee of the Chinese Association of Integrative Medicine, assessed by a study Traditional Chinese Medicine (TCM) doctor; a Zubrod-ECOG performance status of 2 to 4 [43]; willingness to participate in the clinical trial with a comprehensive understanding of the study protocol and the signing of an informed consent form.

Patients undergoing active chemotherapy or radiotherapy were excluded to avoid herb-drug interaction risks. Participants were ineligible if they have acute cardiovascular events, inadequately controlled hypertension, severe liver, renal, or hematopoietic system disorders, or critical conditions endangering their survival. Patients were also excluded if they used antidepressants or anti-anxiety drugs or any form of psychological treatment within one month before enrollment, or participated in any drug clinical trial within three months before participation. The study also excluded women who were pregnant, lactating or were planning to become pregnant.

### Randomization and blinding

Randomization was performed by an independent statistician using SAS 9.4 generating unique codes for Group A (SNS/placebo sequence) or Group B (placebo/SNS sequence). These codes were securely transmitted to Jiangyin Tianjiang Pharmaceutical Company, which packaged identical granules (matched for taste, color, and texture) in sequentially numbered kits.

Both SNS and placebo granules were distributed to study sites by a third-party coordinator uninvolved in recruitment, intervention, or assessment. Participants, clinicians, outcome assessors, and statisticians remained blinded throughout the trial. Emergency unblinding envelopes (containing group assignment) were opened in cases of severe adverse events (AEs).

### Study intervention

**Fig. 1:**
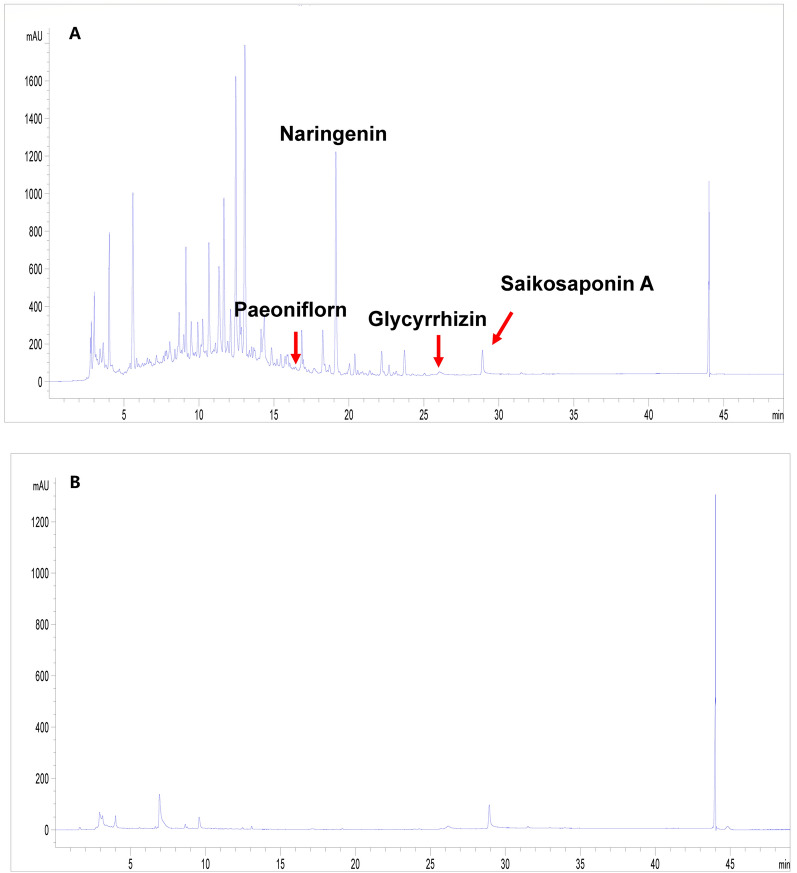
The quality control analysis of SNS and placebo. **A** Representative quality control components of Paeoniflorn, Naringenin, Glycyrrhizin and Saikosaponin A in SNS. **B** The high-performance liquid chromatogram of placebo

Patients were asked to participate over a four-week SNS period, a two-week washout period and a four-week placebo period in different orders, according to their allocated groups. SNS and placebo granules were required to take once a day after meal. SNS granules were manufactured under GMP conditions by Jiangyin Tianjiang Pharmaceutical Co., Ltd. The formulation utilized standardized botanical materials with batch-specific quality verification: *Bupleurum chinense* DC. (Chaihu, #41,200,261, Saikosaponin A ≥ 3.37 mg/g), *Citrus aurantium* L. (Zhishi, #41,010,629.1, Synephrine ≥ 1.26%), *Paeonia lactiflora* Pall. (Baishao, #41,200,048.1, Paeoniflorin ≥ 86.3 mg/g), and *Glycyrrhiza uralensis* Fisch. (Gancao, #41,200,020.2, Glycyrrhizic acid ≥ 28.7 mg/g). Each botanical was dosed at 10 g per package. The matching placebo granules (for taste, smell and color) were produced by the same company and packed in the same designed bags. In addition, the chemical fingerprint of final SNS/placebo granules were determined by the high-performance liquid chromatography (HPLC). Four primary compounds (Saikosaponin A, Naringin, Paeoniflorin, Glycyrrhizic acid) served as chemical standards, and their contents were quantified (Fig. 1). Antidepressants or any form of psychological treatment were not allowed throughout the whole trial. The 2-week washout period in this crossover trial was carefully selected based on prior TCM clinical studies to ensure the time window is longer than five half-lives of herb ingredients and avoid potential herb-drug interactions and individual metabolic variability [44, 45]. Treatment fidelity was monitored using pill counts and patient diaries. Patients were considered adherent if they consumed > 90% of prescribed doses, and those with < 90% adherence were excluded. More details such as monitoring compliance were provided in Supplementary Material 1.3 and 1.4.

### Outcome measurements

The primary outcome measure was the modulation of the Hamilton Depression Scale-24 (HAMD-24) score (assessed by a specialized psychiatrist). Secondary outcome measures encompassed changes in the Functional Assessment of Cancer Therapy—Breast (FACT-B) score and the Traditional Chinese Medicine Syndrome Scale (TCMSS). TCMSS questionnaire was pre-designed and applied to test the benefits of SNS in relieving TCM syndromes of breast cancer patients with MMD, according to the Standard for TCM Diagnostic and therapeutic criteria for disease and pattern (ZY/T001.1-94) published by the Chinese National Administration of Traditional Chinese medicines and the guiding principles for clinical research on TCM new drug (GGTG-2015–12187) published by the Chinese National Medical Products Administration. Depression severity was assessed by psychiatrists using the HAMD-24, quality of life was evaluated with the FACT-B, and TCM syndrome patterns were measured by TCM practitioners using the TCMSS. Safety assessments included liver function tests (LFT) and mental status examination (MSE). All measurements were collected at the baseline, 4th, 6th and 10th weeks in the trial, respectively. Other exploratory efficacy variables included the changes in T lymphocyte subsets, 16S rRNA sequencing, untargeted metabolomics and cytokine array analysis after four weeks treatment. The patients were asked to report changes in medication and potential adverse events at every appointment, and adverse events were graded for severity. (More details about questionnaires were provided in Supplementary Material 1.5).

### Sample size calculation

Referring to the clinical research on the treatment of SNS on depression (Zhang et al., 2022) and the statistical requirements for a 2 × 2 cross-over study [46], the study caculated a total sample size of 42 individuals, with a difference of 1.52 in outcomes, a standard deviation (SD) of 2.9, an alpha level of 0.05, and a power of 0.9. By assuming a conservative 20% drop-out rate, the sample size was adjusted to 54 participants totally. The power analysis was conducted using PASS 15.0.5 (NCSS, USA).

### Sample collection

In this crossover design, paired samples were collected from all participants at baseline and following each 4-week treatment period. Blood samples were collected between 7 and 9 AM after a 12 h overnight fast. Samples were processed within 30 min of collection: centrifuged at 1500 × *g* for 10 min at 4 °C, aliquoted into cryovials, and stored at − 80 °C. Stool samples were collected using Fecal DNA sampling tube (L005, Tianyi, Shanghai, China), immediately frozen at − 80 °C, and processed within 48 h.

### T lymphocyte subsets and analyses

Peripheral blood samples of patients were collected and T lymphocyte subsets were detected by flow cytometry (FACS CANTO II, BD, San Jose, CA, USA). BD Multitest CD3/CD8/CD45/CD4 reagent (340,499, BD, San Jose, CA, USA) was used for the T lymphocyte percentage determinations. Laboratory tests were completed by the clinical laboratory department of Guangdong Provincial Hospital of Chinese Medicine.

### Bacterial DNA extraction, 16S rRNA sequencing, and analyses

DNA extraction from stool samples was performed using the HiPure Stool DNA Kit (Magen, China) with mechanical lysis (0.1-mm zirconia beads, 6 m/s for 40 s), followed by quality verification through Qubit™ dsDNA HS Assay (> 10 ng/μL) and NanoDrop™ (A260/A280 = 1.8–2.0). The V3-V4 regions of 16S rRNA were amplified with primers 341F/806R under 28 PCR cycles, and amplicons were purified with AMPure XP Beads. Libraries were constructed using Illumina DNA Prep Kit (Illumina, CA, USA) and subjected to dual quality control: (1) fragment size 550 ± 50 bp (Agilent 2100 Bioanalyzer), (2) concentration > 2 nM (qPCR). Sequencing was conducted on Illumina NovaSeq 6000 (2 × 250 bp PE, > 50,000 reads/sample), and the result of effective tags was provided in sFig.1. Bioinformatics analysis included: DADA2 R package [47] (version1.14) for ASV inference, RDP classifier [48] (version 2.2) for taxonomy assignment, α/β-diversity analysis in QIIME [49] (version 1.9.1) and R project Vegan package [50] (version 2.5.3), and LEfSe software [51] (version 1.0) for biomarker identification. Sequencing and data analysis services were provided by Guangzhou Kidio Biotechnology Co., Ltd.

### Untargeted metabolomic analysis

The analysis of blood sample metabolites was conducted using an Ultimate U3000 HPLC system (Thermo Fisher Scientific) coupled with a Q Exactive Plus mass spectrometer (Thermo Fisher Scientific). Pooled QC samples were analyzed every 10 injections. Pearson correlation analysis of QC results demonstrated that R-values approaching 1 indicate superior analytical process stability and higher data quality (details in sFig.2). Chromatography was carried out on an Acquity UPLC BEH C18 column (1.7 µm, 100 × 2.1 mm; Waters), with solvent A composed of ultrapure water and solvent B of acetonitrile (CAS 75-05-08; Merck, Germany). The gradient conditions included: 5% solvent B for 0–1 min, a transition to 5–100% solvent B over 19 min, a hold at 100% solvent B for 1 min, and a final return to 5% solvent B for 4 min. The flow rate was set at 200 µL/min, with a column temperature of 37 °C and an injection volume of 5 µL. Mass spectrometer settings were as follows: spray voltage of 2.5 kV in negative ion mode and 3.0 kV in positive ion mode; sheath gas flow of 40; auxiliary gas flow of 10; and sweep cone gas flow of 0. The capillary temperature was maintained at 320 °C. Data acquisition and analysis were performed using Xcalibur 4.0 software (Thermo Fisher Scientific).

Subsequent to raw data acquisition, Compound Discover 3.0 software (Thermo Fisher Scientific) was employed for processing. This involved normalizing the original metabolomics data across batches for comparison. Metabolite identification was based on m/z ratios (± 3 ppm error), retention times, and comparison with a mass spectrometry database. Partial least squares-discriminant analysis (PLS-DA) was conducted using SIMCA^®^ software to enhance visualization. All metabolites reported in this study adhered to the standards set by the Metabolomics Standards Initiative.

### Human cytokine array analysis

Blood sample cytokines were examined using a QAH-CYT-1 antibody array (RayBiotech, USA), following the manufacturer’s guidelines. To measure cytokine secretion levels, 90 μL of concentrated conditioned medium was added to the array, incubated overnight at 4 °C, and then washed with a wash buffer. Biotinylated primary antibodies were added and incubated for 2 h at room temperature, followed by additional washing. Subsequently, Cy3 equivalent dye-conjugated streptavidin was added and incubated for 1 h at room temperature before scanning the array with an InnoScan 300 Microarray Scanner (Innopsys, France). An 8-point standard curve for each target protein was created using array-specific protein standards, and cytokine quantification was based on a standard curve generated from the same array. PCoA analysis, differential heatmap, VENN diagram, correlation lolly plot, and KEGG pathway enrichment analysis were conducted online through BioLadder (bioladder.cn).

### Integration analysis

Microbiome and metabolome statistical correlation analyses were conducted using Spearman’s correlation. The origin‐based metabolic pathway enrichment analysis was performed online in MetOrigin [40] (http://metorigin.met-bioinformatics.cn/). The differential heatmap analysis, the scatter plot and lolly plot statistics were performed online in BioLadder (bioladder.cn) by Spearman’s correlation analysis.

### Statistical analysis

All statistical analyses were conducted using SPSS 26.0 (IBM Corp., Armonk, NY, USA). Two-sided p-values were calculated for all tests, with *P* < 0.05 indicating statistical significance. Only patients who completed the assessments were included in the full analysis set. The distribution of continuous variables was assessed via the Shapiro–Wilk test, while Levene’s test determined the homogeneity of their variances. Continuous data were reported as mean (standard deviation) or median (interquartile range), with categorical data presented as frequencies and percentages. Baseline characteristics were compared across groups using unpaired t tests or the Wilcoxon rank-sum test for continuous variables and χ2 tests or Fisher’s exact tests for categorical variables. For clinical outcomes in crossover design, one-way analysis of variance was employed to evaluate. Multiple comparisons were addressed using distinct approaches: Bonferroni correction for sum score analyses and Benjamini–Hochberg correction for sub-item scores. Exploratory data involved pairwise comparisons, which were analyzed using paired Student’s t tests or the Wilcoxon signed-rank test, and group comparisons were assessed using the unpaired Wilcoxon signed-rank test. Integrated multi-omics analysis used Spearman correlation analysis. Exploratory outcomes were adjusted for multiple comparisons with the Benjamini–Hochberg procedure. The clinical database was developed using EpiData v3.1 software (EpiData Association, Odense, Denmark), and graphs were generated with GraphPad Prism 7.

## Results

### Participant enrollment

**Fig. 2:**
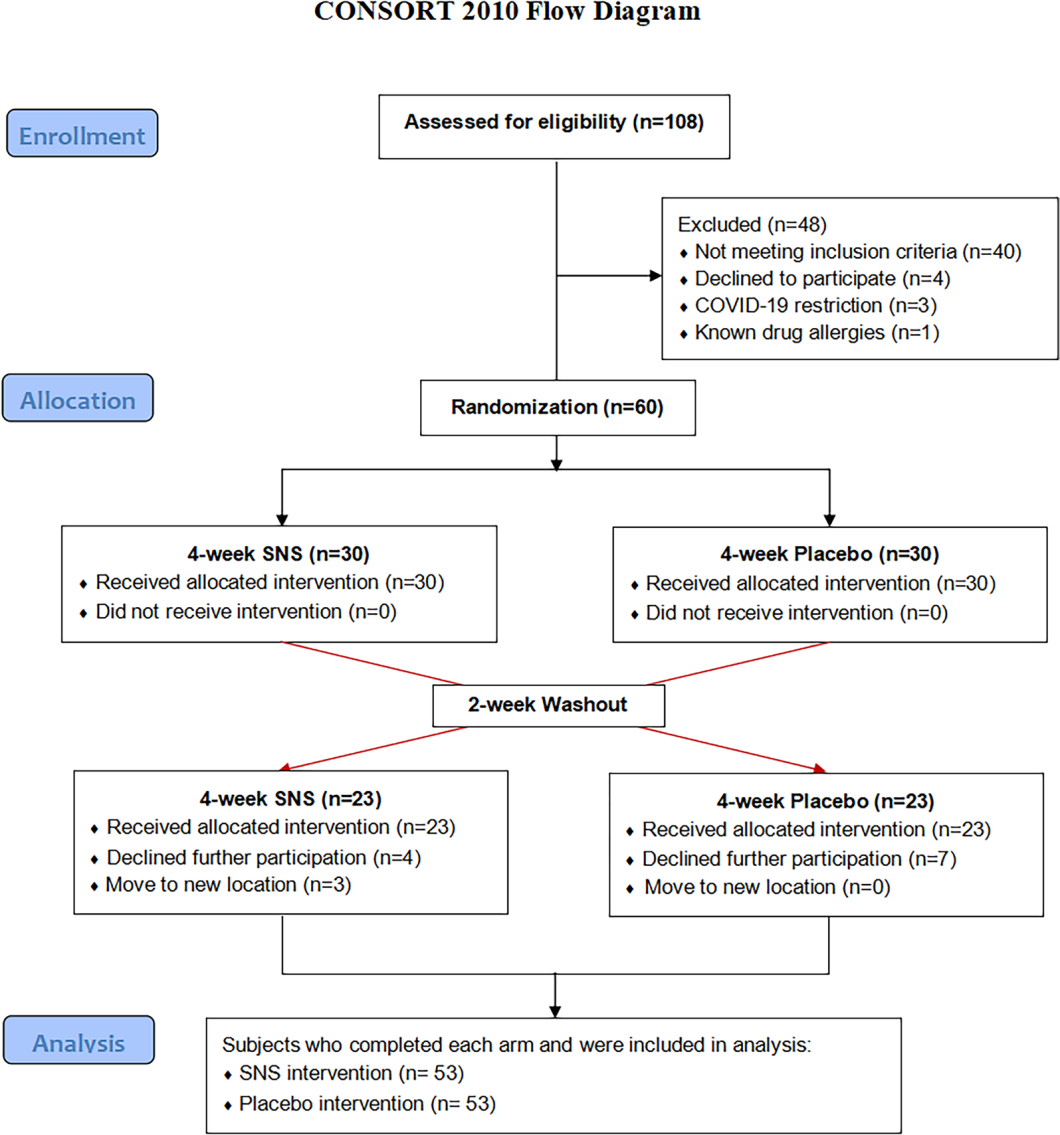
CONSORT 2010 Flow Diagram

From November 7, 2022 to February 22, 2024, 108 breast cancer patients with MMD were recruited from outpatient centers and poster advertisements. After screening for eligibility, a total of 60 breast cancer patients with mild to moderate depression who met the inclusion criteria were enrolled in the study. All patients underwent SNS and placebo treatment in different sequence, with 53 patients completed the SNS treatment and placebo intervention (Fig. 2). There were seven dropout cases in our trial, and the dropout rate was 11.6%.

### Baseline characteristics

The baseline characteristics, including age, marital status, occupation, breast cancer molecular subtype, histological grade, tumor size, lymph node metastasis, distant metastasis, surgical method, history of radiotherapy and chemotherapy, endocrine therapy, and initial depression status, were compared between Group A (SNS/placebo) and Group B (placebo/SNS). No significant statistical differences were observed between the groups (*P* > 0.05) (Table 2).

**Table 2:** Baseline characteristics

Characteristic	All (n = 60)		Group A SNS/Placebo (n = 30)	Group B Placebo/SNS (n = 30)	*P*
Age					
Median (Range), year	50.5(32–75)		53.5 (35–74)	49.0 (32–75)	0.57
< 50 years	28(46.7%)		12	16	0.30
≥ 50 years	32 (53.3%)		18	14	
Marital Status					0.75
Married	58 (96.7%)		29	29	
Divorced	0 (0.0%)		0	0	
Single	2 (3.3%)		1	1	
Widowed	0 (0.0%)		0	0	
Profession					0.90
Mental labour	44(73.3%)		21	23	
Manual labour	4 (6.7%)		2	2	
Retired	11 (18.3%)		7	5	
Pathological classification					0.64
Luminal A	29 (48.3%)		12	17	
Luminal B	16 (26.7%)		9	7	
Her-2	8 (13.3%)		5	3	
TNBC	7 (11.7%)		4	3	
Histological grade					0.36
1	9 (15.0%)		4	5	
2	40 (66.7%)		18	22	
3	11(18.3%)		7	4	
TNM stage					0.41
I	33 (55.0%)		19	14	
II	19 31.7%)		7	14	
III	8 (13.3%)		5	3	
Tumor size					0.59
< 2 cm	30(50.0%)		14	16	
2–5 cm	21 (35.0%)		11	10	
≥ 5 cm	9 (15.0%)		5	4	
Number of metastatic lymph nodes					1.00
0	27(45.0%)		12	15	
1–3	31 (51.7%)		17	14	
≥ 4	2 (3.3%)		1	1	
Tumor distant metastasis					ns
Presence	0 (0.0%)		0	0	
Absence	60(100.0%)		30	30	
Surgical approach					0.58
Total mastectomy	40(66.7%)	21		19	
Breast-conserving surgery	20 (33.3%)	9		11	
Prior radiotherapy					1.00
Use	38(63.3%)	19		19	
Unuse	22 (36.7%)	11		11	
Prior chemotherapy					1.00
Use	30 (50.0%)	15		15	
Unuse	30 (50.0%)	15		15	
Current/prior endocrinotherapy					
SERM					0.12
Use	24(40.0%)	18		12	
Unuse	36 (60.0%)	12		18	
AI					0.44
Use	33 (55.0%)	15		18	
Unuse	27 (45.0%)	15		12	
OFS					0.69
Use	7(11.7%)	4		3	
Unuse	53(88.3%)	26		27	
SERD					0.75
Use	2 (3.3%)	1		1	
Unuse	58 (96.7%)	29		29	
Depression status					
Mild	31(51.7%)	15		16	0.80
Moderate	29(48.7%)	15		14	

*SERM* Selective estrogen receptor modulators, *AI* aromatase inhibitors, *OFS* Ovarian function suppression, *SERD* Selective estrogen receptor degraders

### SNS reduces HAMD-24 scores in breast cancer patients with MMD

**Fig. 3:**
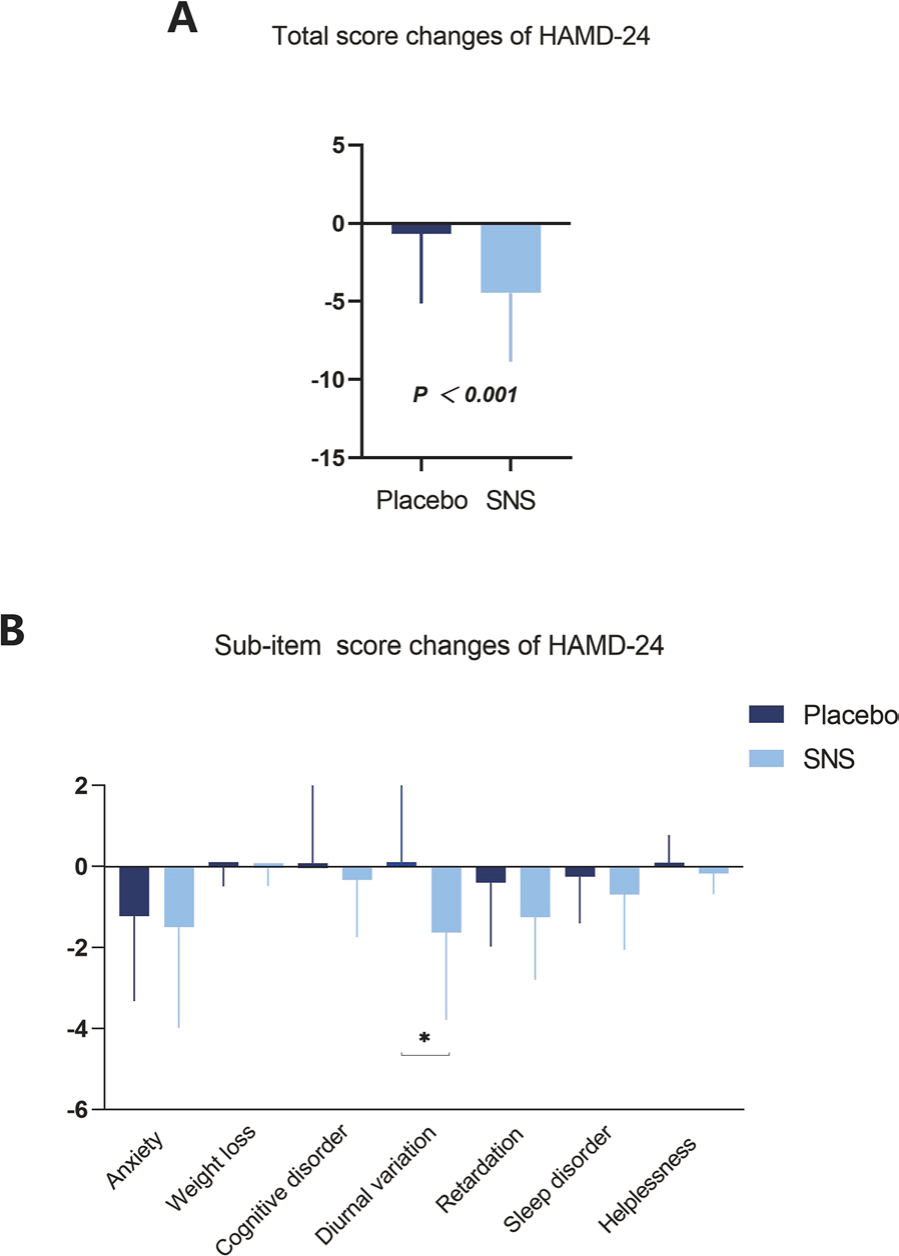
SNS significantly reduces the depression score evaluated by HAMD-24 scale. **A** Total score changes of HAMD-24 scale following SNS and placebo interventions. **B** Sub-item score changes of HAMD-24 scale. The data are expressed as Mean ± SD (95% CIs) and analyzed by one-way analysis of variance. *P_*adjust*_ < 0.05

To determine the effects of SNS improving the depression status of breast cancer patients, the HAMD-24 scale score was detected as the primary outcome between the SNS treatment and placebo intervention. Compared to placebo intervention, HAMD-24 scores of SNS treatment were significantly reduced (− 4.46 ± 4.40 (95% CI − 5.69, − 3.24) *vs. -*0.66 ± 4.46 (95% CI − 1.89, 0.57, *P*_adjust_ < 0.001) (Table 3 & Fig. 3A). In addition, the HAMD-24 scale comprises seven indexes: anxiety, weight loss, cognitive disorder, diurnal variation, retardation, sleep disorder, and helplessness. Considering each index, SNS administration significantly improved the scores for the diurnal variation (− 1.62 ± 2.17 (95% CI − 2.22, − 1.01) *vs.* 0.11 ± 1.97 (95% CI − 0.43, 0.66, *P*_adjust_ < 0.001) (Table 3 & Fig. 3B). These results provide the definitive evidence supporting the positive therapeutic value of SNS in relieving the depression degree of breast cancer patients.

**Table 3:** Changes of HAMD-24 score after 4-Week treatment with SNS or placebo

Outcomes	Placebo (n = 53)			SNS (n = 53)					
	Mean	SD	95%CI	Mean	SD	95%CI	F	*P*	*P *_*adjust*_
HAMD-24	− 0.66	4.46	− 1.89–0.57	− 4.46	4.40	− 5.69 to − 3.24	15.92	< 0.001	< 0.001
Anxiety	− 1.23	2.09	− 1.80–0.65	− 1.50	2.49	− 2.19 to − 0.81	0.04	0.85	0.91
Weight loss	− 0.02	0.47	− 0.09–0.24	− 0.04	0.44	− 0.16 to 0.08	1.71	0.20	0.37
Cognitive disorder	0.08	2.01	− 0.63–0.48	− 0.33	1.41	− 0.72 to 0.07	0.63	0.43	0.59
Diurnal variation	0.11	1.97	− 0.43–0.66	− 1.62	2.17	− 2.22 to − 1.01	15.44	< 0.001	< 0.001
Retardation	− 0.40	1.57	− 0.83–0.04	− 1.25	1.55	− 1.68 to − 0.82	6.44	0.02	0.06
Sleep disorder	− 0.26	1.15	− 0.58–0.05	− 0.69	1.37	− 1.07 to − 0.31	2.91	0.10	0.24
Helplessness	0.09	0.69	− 0.09–0.28	− 0.17	0.51	− 0.32 to − 0.03	3.65	0.06	0.19

Values are presented as mean ± SD (95% CIs). Adjusted for multiple comparisons using the Holm-Bonferroni procedure

### SNS improves the quality of life for breast cancer patients with MMD

**Fig. 4:**
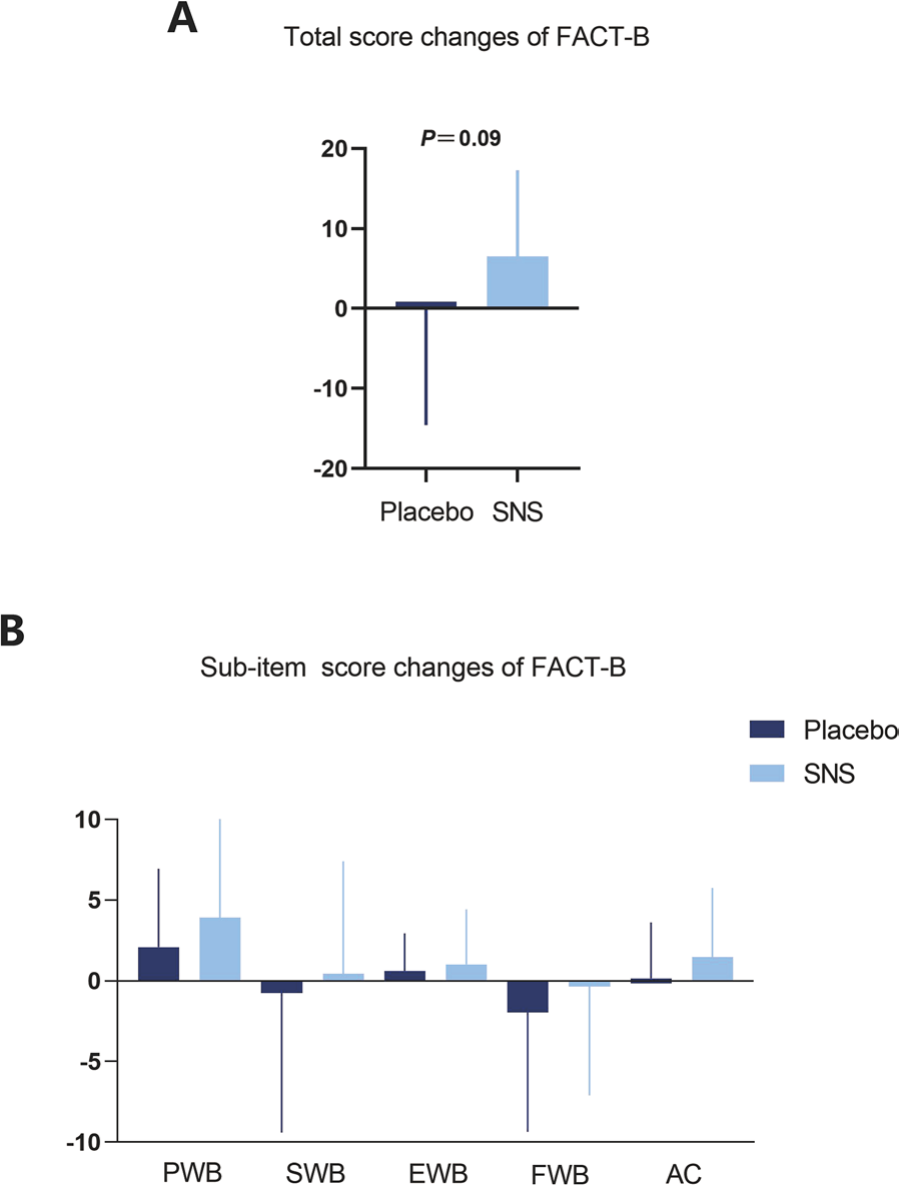
SNS significantly improves the quality of life evaluated by FACT-B scale. **A** Total score changes of FACT-B scale following SNS and Placebo Interventions. **B** Sub-item score changes of FACT-B scale. The data are expressed as Mean ± SD (95% CIs) and analyzed by one-way analysis of variance. * P_*adjust*_ < 0.05

Depression is closely correlated with quality of life of breast cancer patients, and TCM has long been appreciated for its priority in improving quality of life of cancer patients. Therefore, the FACT-B questionnaire was applied to test the benefits of SNS in improving the life quality of breast cancer patients with MMD. Compared to placebo intervention, FACT-B scores of SNS treatment were increased (6.50 ± 10.79 (95% CI 3.50, 9.50) *vs. *− 0.15 ± 14.45 (95% CI − 3.83, 4.13, *P*_adjust_ = 0.09) (Table 4 & Fig. 4A). In addition, the FACT-B scale can be divided into five sub-items including physical well-being (PWB), social well-being (SWB), emotional well-being (EWB), functional well-being (FWB) and additional concerns (AC) (Table 4 & Fig. 4B). These finding demonstrate that SNS treatment results in a better quality of life, which might be closely associated with the improved depression status.

**Table 4:** Changes of FACT-B score after 4-Week treatment with SNS or placebo

Outcomes	Placebo (n = 53)			SNS (n = 53)					
	Mean	SD	95%CI	Mean	SD	95%CI	F	*P*	*P *_*adjust*_
FACT-B	− 0.15	14.45	− 3.83–4.13	6.50	10.79	3.50–9.50	5.20	0.03	0.09
PWB	2.09	4.85	0.76–3.43	3.90	6.58	2.07–5.74	1.67	0.20	0.34
SWB	− 0.75	8.66	− 3.32–1.29	0.44	6.97	− 1.95–1.75	0.50	0.49	0.61
EWB	0.62	2.31	− 0.02–1.26	1.02	3.40	0.07–1.97	0.33	0.57	0.66
FWB	− 1.96	7.40	− 4.00–0.08	− 0.35	6.76	− 2.23–1.53	0.65	0.43	0.64
AC	0.15	3.46	− 0.80–1.10	1.48	4.28	0.29–2.67	2.37	0.13	0.28

Values are presented as mean ± SD (95% CIs)

Adjusted for multiple comparisons using the Holm-Bonferroni procedure

*PWB* physical well-being, *SWB* social well-being, *EWB* emotional well-being, *FWB* functional well-being, *AC* additional concerns

### SNS improves the TCM syndromes for breast cancer patients with MMD

**Fig. 5:**
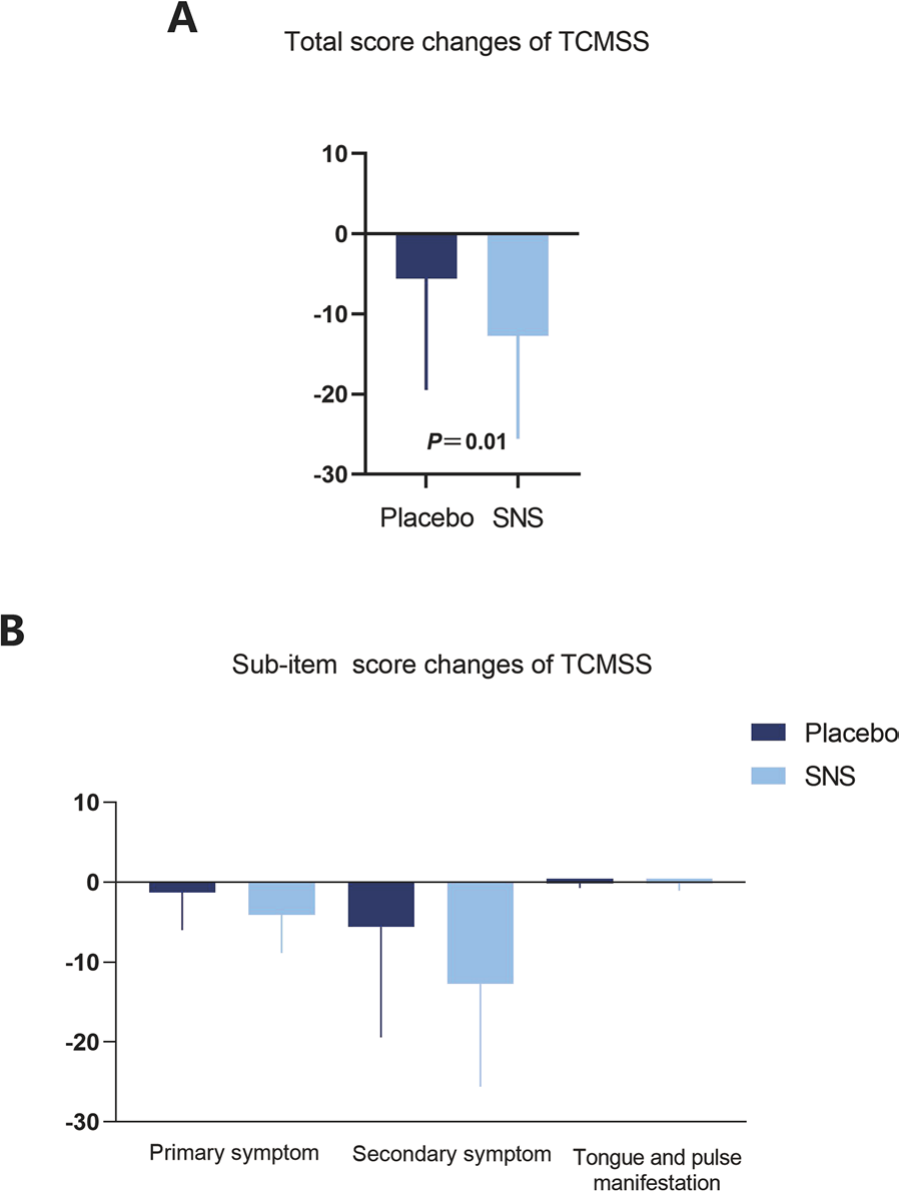
SNS significantly reduces the TCM syndromes score evaluated by TCMSS scale. **A** Total score changes of TCMSS scale following SNS and placebo interventions. **B** Sub-item score changes of TCMSS scale. The data are expressed as Mean ± SD (95% CIs) and analyzed by one-way analysis of variance. * P_*adjust*_ < 0.05

Since TCM prescriptions are created considering patients’ syndromes, the evaluation of symptom changes after SNS treatment is crucial to determine its clinical efficacy. Compared to placebo intervention, TCMSS scores of SNS treatment were decreased (− 12.71 ± 12.88 (95% CI − 16.30, − 9.13) *vs.* − 5.60 ± 13.88 (95% CI − 9.42, − 1.79), *P*_adjust_ = 0.01) (Table 5 & Fig. 5A). In addition, the TCMSS scale consisted of three sub-items including primary symptom, secondary symptom and tongue and pulse manifestation. The sub-item evaluation showed that SNS intervention improved primary symptom scores (− 4.08 ± 4.77 (95% CI − 5.40, − 2.75) *vs.* − 1.28 ± 4.69 (95% CI − 2.58, − 0.01, *P*_adjust_ = 0.10) and secondary symptom scores (− 8.44 ± 9.49 (95% CI − 11.08, − 5.80) *vs.* − 4.38 ± 10.76 (95% CI − 7.34, − 1.41, *P*_adjust_ = 0.15) (Table 5 & Fig. 5B). These finding demonstrate that SNS treatment can significantly ameliorate TCM syndromes, which contribute to the improved depression score and quality of life.

**Table 5:** Changes of TCMSS score after 4-Week treatment with SNS or placebo

Outcomes	Placebo (n = 53)			SNS (n = 53)					
	Mean	SD	95%CI	Mean	SD	95%CI	F	*P*	*P *_*adjust*_
TCMSSS	− 5.60	13.88	− 9.42 to − 1.79	− 12.71	12.88	− 16.30 to − 9.13	9.43	0.004	0.01
Primary symptom	− 1.28	4.69	− 2.58 to 0.01	− 4.08	4.77	− 5.40 to − 2.75	6.70	0.01	0.10
Secondary symptom	− 4.38	10.76	− 7.34 to − 1.41	− 8.44	9.49	− 11.08 to − 5.80	4.22	0.04	0.15
Tongue and pulse manifestation	− 0.15	0.57	− 0.31 to 0.01	− 0.15	0.89	− 0.40 to 0.10	0.001	0.97	0.97

Values are mean ± SD (95% CIs). Adjusted for multiple comparisons using the Holm-Bonferroni procedure

### Safety evaluation and adverse events

To ensure the safety of drug treatment, LFT and MSE were systematically conducted at baseline, 4th week, 6th week, and 10th week for each participant. Our analyses indicated that either SNS or placebo treatment brought little influence on liver function and mental status (sTable 1).

Throughout the study, we recorded only two mild adverse events: one case of transient diarrhea in the SNS group (resolved within 48 h without intervention) and one case of dry mouth in the placebo group (managed with increased fluid intake). Both events were classified as Grade 1 according to CTCAE criteria and did not lead to treatment discontinuation. The complete adverse event profile was presented in sTable 2. Collectively, these findings suggest that four weeks SNS treatment against depression in breast cancer patients is responsive with high safety.

### Gut microbiota analysis of SNS in breast cancer patients with MMD

**Fig. 6:**
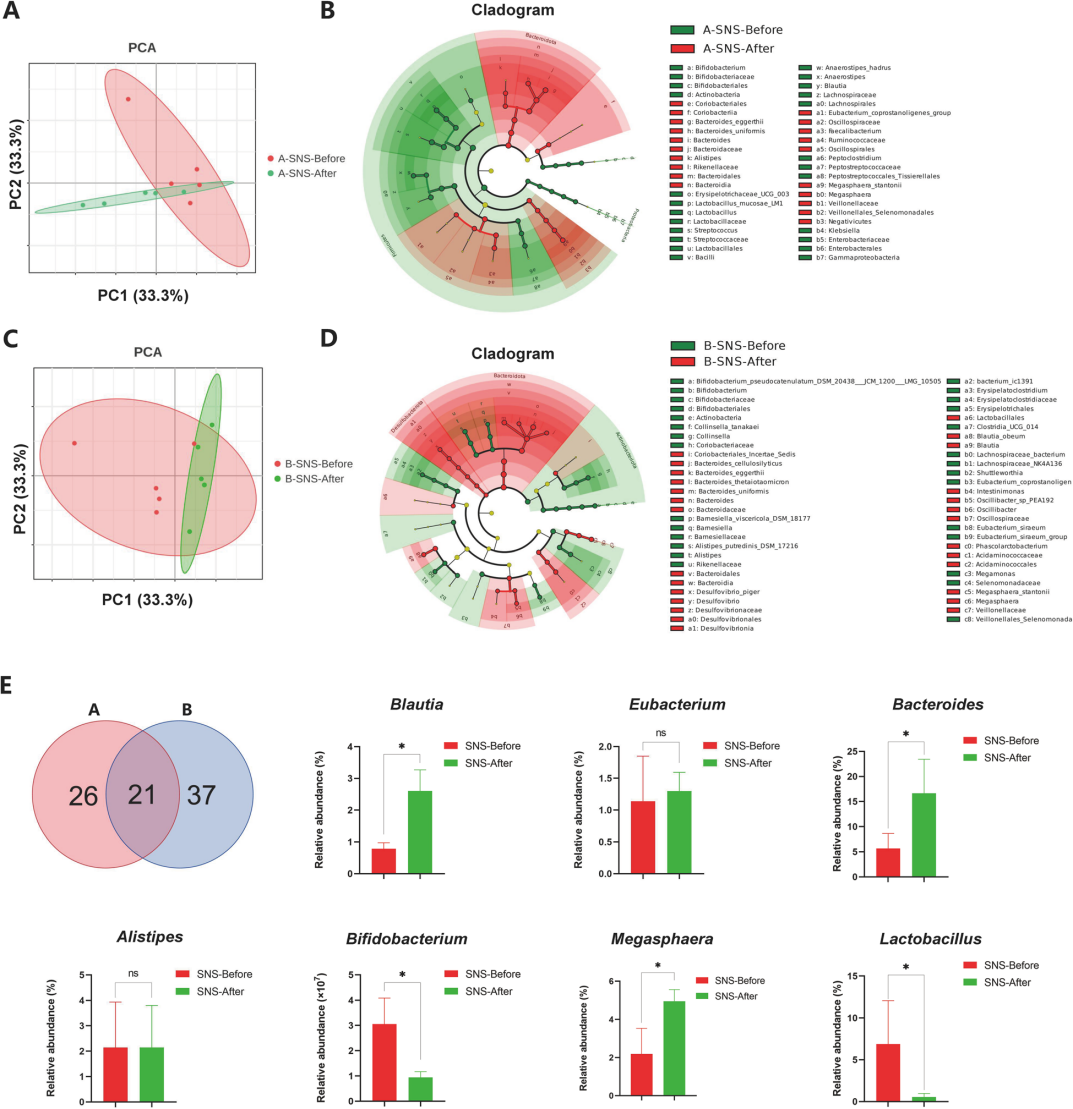
Effects SNS on the gut microbiota of breast cancer patients with MMD. **A** β diversity changes following SNS intervention before and after treatment in Groups A (n = 5). **B** LEfSe analysis of SNS intervention before and after treatment in Groups A (n = 5). The LDA cut-off is 4. **C** β diversity changes following SNS intervention before and after treatment in Groups B (n = 5). **D** LEfSe analysis of SNS intervention before and after treatment in Groups B (n = 5). The LDA cut-off is 4. **E** Venn analysis and effects of SNS on specific bacterial genus (n = 10). The data are expressed as Mean ± SD and a analyzed by paired Student’s t-test or Wilcoxon signed-rank test. * P_*adjust*_ < 0.05

To investigate whether the clinical efficacy of SNS is related to the gut microbiota, we conducted 16S rRNA microbial sequencing on stool samples of five participants before and after SNS intervention from two groups. Based on MetaPhlAn2 taxonomic analysis, the α-diversity results (ACE & Shannon index) showed the differences between the pre- and post-intervention with SNS (sFig. 3). In terms of β-diversity, the PCoA analysis also demonstrated significant differences in microbial composition before and after SNS intervention (Fig. 6A, C). Therefore, we employed LEfSe analysis to further identify the key differential bacterial species involved in. The results (Fig. 6B, D) revealed that there were 47 differential bacteria in Group A and 58 in Group B before and after SNS intervention (LDA score ≥ 4), and 21 kinds of bacterial were overlapped between the two groups after Venn analysis (Fig. 6E). Subsequently, we comprehensively analyzed the abundance changes in the top seven differential bacterial species (LDA score ≥ 9) shared by both Groups A and B. The data demonstrated that following SNS intervention, there were notable increases in the abundance of *Blautia, Bacteroides,* and *Megasphaera (P*_adjust_ < 0.05), while *Bifidobacterium* and *Lactobacillus* exhibited significant reduction *(P*_adjust_ < 0.05). No statistically significant differences were observed in the abundance changes of *Eubacterium* and *Alistipes*. In conclusion, SNS intervention appears to exert beneficial effects on breast cancer patients with MMD by influencing the gut microbiota, particularly for *Blautia*, *Bacteroides*, *Megasphaera*, *Bifidobacterium*, and *Lactobacillus*.

### Serum metabolomic analysis of SNS in breast cancer patients with MMD

**Fig. 7:**
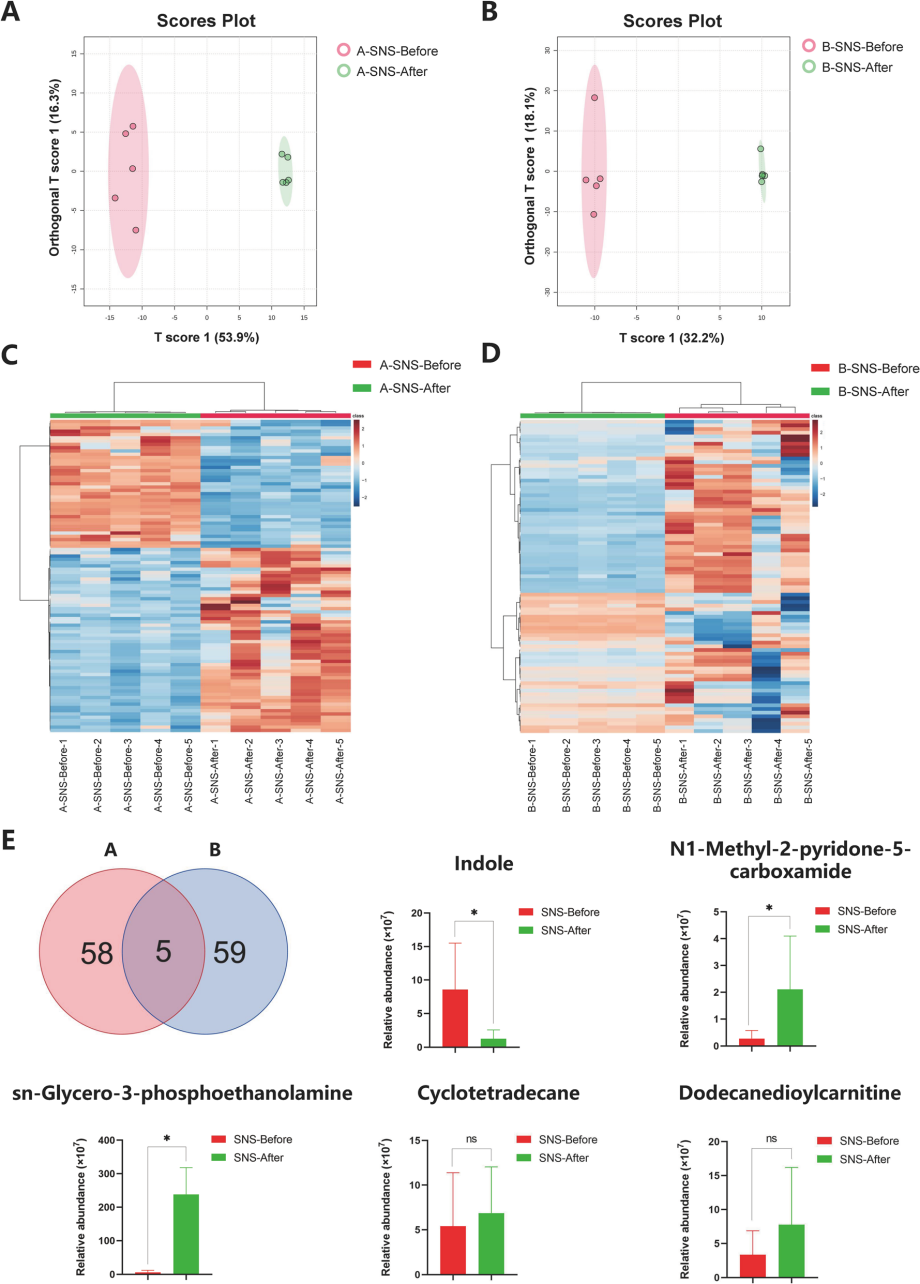
Influence of SNS on serum metabolomic of breast cancer patients with MMD. **A** and **B** PLS-DA analysis of serum metabolites before and after SNS treatment in Groups A and B (n = 5). **C** and **D** Heatmap analysis of differential metabolites in group A and B after SNS treatment (n = 5). **E** Venn analysis and effects of SNS on specific metabolites (n = 10). The data are expressed as Mean ± SD and a analyzed by paired Student’s t-test or Wilcoxon signed-rank test. * P_*adjust*_ < 0.05

In order to investigate the impact of SNS on serum metabolites, the UPLC-MS/MS-based metabolomics was performed. A total of 725 metabolites in serum were successfully identified. To differentiate the metabolomic profiles among different groups, we conducted clustering analysis using PLS-DA method, the results demonstrated a clear separation between the pre- and post-treatment serum after SNS treatment, indicating a significant alteration in the metabolite profiles of breast cancer patients with MMD (Fig. 7A, B). Furthermore, we compared the differential metabolites between the pre- and post-treatment serum by heatmap analysis (VIP score > 1.15 & *P*_adjust_ < 0.05). The results revealed a substantial modulation exerted by SNS on certain metabolites, presented by increased expression of N1-Methyl-2-pyridone-5-carboxamide, sn-Glycero-3-phosphoethanolamine, and tetradecanoic acid. Meanwhile, a concurrent decrease in the levels of indole, isoleucine, and cyclotetradecane was also found. Notably, a similar pattern was observed in group B (Fig. 7C, D). These findings revealed that SNS administration brought distinct metabolic alterations in patients’ serum.

To identify the key metabolites concurrently changed in both groups, the Venn diagram analysis was conducted and showed that five differential metabolites were shared by both groups (Fig. 7E). We subsequently conducted a comprehensive analysis of the identified metabolites before and after SNS intervention. The results indicated that after SNS treatment, the level of indole was significantly decreased *(P*_adjust_ < 0.05), and the levels of N1-Methyl-2-pyridone-5-carboxamide and sn-Glycero-3-phosphoethanolamine levels showed a significant increase *(P*_adjust_ < 0.05), while no significant difference was found in the levels of cyclotetradecane and dodecanedioylcarnitine. These findings suggest that these three key differential metabolites may be highly correlated with the effects of SNS in relieving depression of breast cancer patients.

### Effects of SNS on serum cytokine expression in breast cancer patients with MMD

**Fig. 8:**
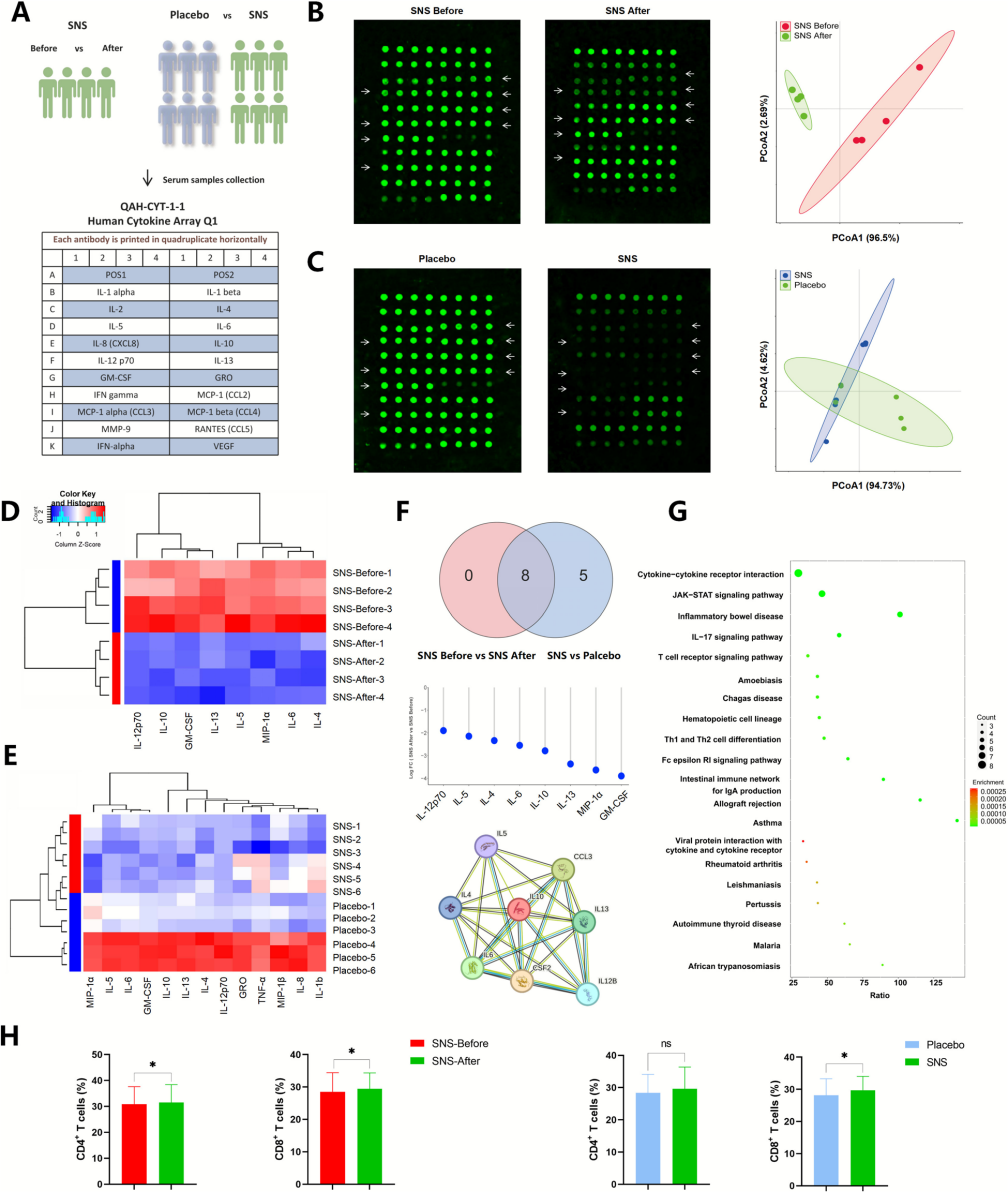
Influence of SNS on serum cytokine expression in breast cancer patients with MMD. **A** Graphic description of two cohorts’ comparison and detailed information of human cytokine array map. **B** Representative cytokines expression and PCoA analysis in patients treated with SNS before and after (n = 4). **C** Representative cytokines expression and PCoA analysis in patients treated with placebo and SNS, respectively (n = 6). **D** Heatmap analysis of differential cytokines before and after SNS intervention. **E** Heatmap analysis of differential cytokines between placebo and SNS treatment groups. **F** Venn, relative changes and PPI network analysis of overlapped differential human cytokines in two cohorts. **G** KEGG enrichment of potential molecular pathways. **H** Changes of CD4^+^ and CD8^+^ T populations following SNS and placebo treatment (n = 53 & n = 46, respectively). Data are expressed as Mean ± SD, pairwise comparisons were analyzed using analyzed by paired Student’s t-test or Wilcoxon signed-rank test, and group comparison was analyzed by unpaired Wilcoxon signed-rank test. * *P*_*adjust*_ < 0.05

To investigate the modulatory effects of SNS on improving immunity of breast cancer patients, human cytokine array tests were performed by using serum samples from both groups (Fig. 8A). PCoA analysis demonstrated a clear separation between the pre- and post-treatment serum after SNS treatment (Fig. 8B), as well as the placebo and SNS intervention groups (Fig. 8C), indicating SNS brought a significant alteration in the cytokine profiles of breast cancer patients with MMD. In addition, heatmap analysis (| FC |> 1 & *P*_adjust_ < 0.05) showed that SNS resulted in a remarkable reduction of several cytokines (Fig. 8D, E). The Venn analysis revealed that there were eight overlapped cytokines affected by SNS in both groups, including MIP-1a, GM-CSF, IL-13, IL-6, IL-10, IL-5, IL-12p70, and IL-4; and the relative changes in these cytokines are presented (Fig. 8F). Previous studies have revealed that MIP-1a, GM-CSF, IL-13, IL-6, IL-10, IL-5, IL-12p70, IL-4 were all associated with cognitive performance [40, 41]. Meanwhile, the PPI network analysis indicate a strong correlation among cytokines, and IL-10 acted as the core factor. Additionally, Kyoto Encyclopedia of Genes and Genomes (KEGG) analysis (Fig. 8G) showed enrichment of cytokine–cytokine receptor interaction, JAK-STAT signaling pathway, inflammatory bowel disease and T cell receptor signaling pathways, suggesting that the T cell immunity may be affected by SNS. Therefore, T lymphocyte subsets in the peripheral blood samples of enrolled patients were further analyzed, including the population of CD4^+^ T cells and CD8^+^ T cell. Notably, a significant elevation of CD8^+^ T cell after SNS administration was found in two comparisons (*P*_adjust_ < 0.05), and a remarkable CD4^+^ T elevation was also observed in patient before-after comparison (*P*_adjust_ < 0.05) (Fig. 8H). Collectively, these results indicate that the antidepressant effects of SNS might be correlated with the improved immunity, and highlight a potential link between the gut microbiota, serum metabolites and cytokine regulation.

### Integrated multiomics analysis of SNS in improving depression of breast cancer

**Fig. 9:**
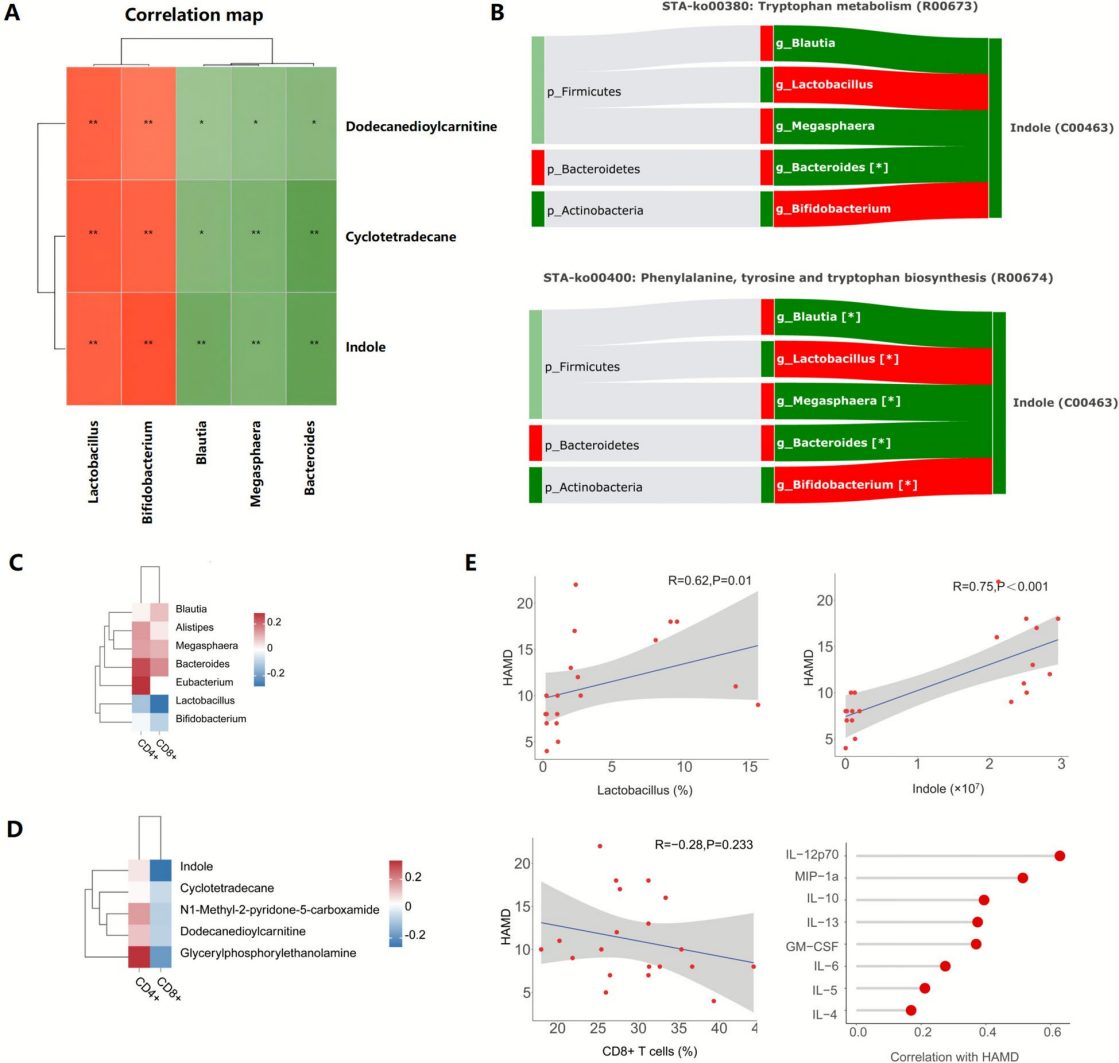
Integrated multiomics analysis and potential mechanism of SNS in improving depression of breast cancer. **A** Correlations between the key differential bacterial genera and metabolites. The red and green indicate the positive and negative correlation, respectively. **B** Deep MetOrigin Analysis for correlations between the key differential bacterial species and indole. The dark red/green bars indicate significantly up/down regulated bacterial genera (| FC |> 1, *P*_*adjust*_ < 0.05), the dark red/green bands indicate significant positive/negative correlation (| R |> 0, *P*_*adjust*_ < 0.05). **C** Correlations between key differential bacterial genera and T lymphocytes. The red and blue indicate the positive and negative correlation, respectively. **D** Correlations between key differential metabolites and T lymphocytes. The red and blue indicate the positive and negative correlation, respectively. **E** Spearman correlation analysis between *Lactobacillus*, indole, CD8^+^ T cell, key differential human cytokines and HAMD scores. The regression line is blue, and the shading indicates the 95% confidence interval. n = 20, * P_*adjust*_ < 0.05

Following the significant impact of SNS intervention on T lymphocyte subsets, gut microbiota, and metabolomics in depressed breast cancer patients, we conducted an integrated analysis to delve deeper into the potential mechanisms underlying the actions of SNS. To clarify the interplay between gut microbiota and serum metabolites, we conducted a Spearman correlation analysis of seven key differential bacterial genera and five key differential metabolites. The results (Fig. 9A) revealed a positive correlation (*P*_adjust_ < 0.05) between the metabolites Cyclotetradecane, Dodecanedioylcarnitine, Indole, and the genera *Bifidobacterium* and *Lactobacillus*, while showing a negative correlation (*P*_adjust_ < 0.05) with *Alistipes*, *Blautia*, and *Bacteroides*. Additionally, the DMOA analysis (Fig. 9B) underlined the potential role of key differential bacterial genera in the pharmacological mechanism of SNS, and established a robust link between the microbial community and the metabolite indole. Notably, it suggested two pathways may be influenced by the key bacterial genera and metabolites, including pathway ko00400 (Phenylalanine, tyrosine and tryptophan biosynthesis) and pathway ko00380 (Tryptophan metabolism), highlighting tryptophan metabolism may be the potential target of SNS (*P*_adjust_ < 0.05).

Subsequently, we conducted Spearman correlation analysis (Fig. 9C) between the key differential bacterial genera, differential metabolites, and the proportions of T lymphocyte subsets. The results showed a negative correlation between *Lactobacillus* with the proportion of CD8^+^ T cells, and a positive correlation between sn-Glycero-3-phosphoethanolamine and the proportion of CD4^+^ T cells. Furthermore, the Spearman correlation analysis (Fig. 9D) between the key differential metabolites and T lymphocyte subsets showed that CD8^+^ cells also have a negative correlation with indole.

Given the potential correlation between *Lactobacillus*, indole, and CD8^+^ T cells, their contribution to the beneficial effect of SNS on MMD breast cancer patients deserved further investigation. Therefore, we conducted Spearman correlation analysis (Fig. 9E) between the abundance of *Lactobacillus*, indole levels, the proportion of CD8^+^ T cells and the differential cytokines with the patients’ HAMD depression score from clinical trial. The results demonstrated a positive correlation between the abundance of *Lactobacillus* and HAMD score (*R* = 0.62, *P*_adjust_ = 0.01), a positive correlation between indole levels and HAMD score (*R* = 0.75, *P*_adjust_ < 0.001), and a negative correlation between the proportion of CD8^+^ T cells and HAMD score (*R* = − 0.28, *P*_adjust_ = 0.223), and a positive correlation between cytokines quantity of MIP-1a, GM-CSF, IL-13, IL-6, IL-10, IL-5, IL-12p70, IL-4 and HAMD score. Notably, these results based on correlation analysis from small samples can only contribute to hypothesis generation rather than confirmatory findings. Collectively, these findings suggest the potential value of the *Lactobacillus*-Indole-CD8^+^ T cell axis as a signaling responsible for depression occurrence and prognosis in breast cancer patients, which deserves future experimental validation to confirm mechanistic links.

## Discussion

In this study, we demonstrated that SNS is efficacious and safe in treating MMD among breast cancer patients, improving quality of life and TCM syndrome scores, with the *Lactobacillus*-Indole-CD8^+^ T axis playing a potential role in the pharmacological activity of SNS. Previously, a systematic review of seven randomized trials showed that SNS may be more effective in reducing HAMD scores than fluoxetine hydrochloride after treatment for either four or eight weeks [38]. Although SNS is commonly prescribed to treat depression in clinic, its influence on breast cancer is still limited. Our previous findings reported that SNS could improve the depressive behaviors of mice bearing breast cancer, as well as inhibiting breast cancer growth and metastasis through modulating estrogen metabolism and heat shock protein expression [39, 41]. However, the clinical effectiveness of SNS on breast cancer patients is unknown. This study presents the first clinical evidence supporting the application of SNS for MMD treatment in breast cancer patients. Notably, the improved quality of life (FACT-B scores) and TCM syndromes (TCMSS scores) might be potentially associated with the alleviation of depressive symptoms following SNS treatment. Evidence has shown that mental disorders significantly impact daily activities, overall health, cognitive function, and employment status, as well as impeding workplace productivity, which highlights the crucial role of alleviating depressive symptoms in enhancing the quality of life [52]. Moreover, SNS administration did not bring blood toxicity and dysfunctions of liver, indicating the high safety of SNS for breast cancer patients. However, the current study only enrolled breast cancer patients in the consolidation phase, whether SNS could reach the similar clinical benefits in the phases of diagnosis, surgery or chemotherapy worth future investigation. Besides, the influence of pathological subtype of breast cancer on the clinical effects of SNS is interesting to explore.

Increasing preclinical and clinical studies show that dysbiosis, or dysregulation of the gut microbiota, may contribute to depression’s onset and progression [53]. An eight-week RCT found that daily multi-strain probiotic treatment significantly relieved depressive symptoms and was acceptable, well-tolerated, and safe in patients with MMD [54]. In our study, the improvement in depression symptoms was also accompanied by the regulation of gut microbes and microbial metabolites. After SNS treatment, a notable reduction in the abundance of *Lactobacillus* species was observed, along with a positive correlation with HAMD scores. Interestingly, although *Lactobacill*us has been recognized as a “psychotropic probiotic” in several clinical trials [55, 56], some clinical research has indicated that the common probiotic preparations (consisting of *Bifidobacterium* spp. and *Lactobacillu*s spp.), did not significantly improve symptoms of mental disorders [57]. A meta-analysis has also indicated that probiotic supplementation has minor effect on mood regulation [58]. Notably, *Streptococcus*, *Lactobacillus*, and *Eggerthella* genera had been found enriched in psychiatric patients, and correlated with more severe clinical symptoms [59]. Moreover, A recent study revealed that *Lactobacilli* can produce indole from tryptophan in foods, thereby activating the aryl hydrocarbon receptor (AhR) on tumor-associated macrophages (TAMs), leading to the reduction of interferon secreted by CD8^+^ T cells, and finally promoting the growth of pancreatic ductal adenocarcinoma [60]. These findings, together with our results, challenges our inherent impression of *Lactobacillus*, which is not always beneficial. It also suggests that the traditional “probiotic” concept needs to be reexamined and verified. The effects of *Lactobacillus* appear to be influenced by different conditions, such as the degree of depression or the phase of cancer. Equally important, the expression level of *Lactobacillus* in breast cancer patient with depression is currently unclear and requires large-scale clinical testing for verification.

Metabolism plays a crucial role in mediating the effects of gut microbiota on depressive behaviors, as well as on cancer prognosis [61]. Following SNS administration, a notable alteration in the metabolite profiles of breast cancer patients with MMD was detected. Here in, a substantial reduction in the level of indole was observed after SNS administration, whereas the levels of N1-Methyl-2-pyridone-5-carboxamide and sn-Glycero-3-phosphoethanolamine showed a marked increase. Notably, we identified a correlation between the abundance of *Lactobacillus* and indole levels, along with a positive correlation with depression scores. Indole is a typical nitrogen-containing aromatic heterocyclic compound primarily involved in the metabolism of tryptophan. The tryptophan metabolism pathway is primarily divided into three distinct routes including the kynurenine pathway, the 5-hydroxytryptamine (serotonin) pathway, and the indole pathway. The indole pathway mainly involves the metabolism of tryptophan by the gut microbiota to indole, which is then converted into derivatives such as indole acetic acid (IAA) and indolepropionic acid (IPA) [62]. While the biological functions of indole derivatives remain to be fully elucidated, there is growing interest in their potential role in the pathogenesis of depression. Indole and indole derivatives produced by intestinal microbes may negatively affect brain excitability in both human and animal models [63–65]. Interestingly, recent research has indicated that indole and its derivatives constitute the primary metabolites synthesized by *Lactobacillus reuteri,* indicating a positive correlation between indole and *Lactobacillus* [66]. Furthermore, indole and their derivatives can act as endogenous ligands to activate AhR, a poor prognosis marker of breast cancer [67]. Besides, the kynurenine and 5-hydroxytryptamine pathways were also highlighted in facilitating breast cancer growth and metastasis. Therefore, tryptophan metabolism has become a significant therapeutic target for improving cancer prognosis and related depression. It is promising to develop early warning risk model based on tryptophan metabolism for breast cancer patients in the future.

It has been reported that cytokine dysregulation is one of hallmarks of depression, as well as malignancies [9]. Specifically, we observed a significant decrease in the levels of MIP-1α, GM-CSF, IL-13, IL-6, IL-10, IL-5, IL-12p70, and IL-4 after SNS administration, all of which are associated with the severity of MMD symptoms and cancer prognosis. For example, changes in IL-6 serum levels have been reported as one of the most consistent abnormalities in both depression and cancer [68]. In breast cancer, IL-6 promotes breast cancer cell viability, proliferation, and angiogenesis [69]. Meanwhile, a population-based longitudinal study indicated that elevated IL-6 level in childhood was associated with an increased risk of depression [70], and a systematic review also revealed that patients with melancholic depression exhibit higher peripheral IL-6 level compared to controls [71]. Mechanistically, IL-6 has been shown to directly influence serotonin transporter (SERT) levels and subsequent serotonin reuptake [72]. GM-CSF, another cytokine, is known to facilitate the development of the immunosuppressive breast cancer microenvironment by regulating myeloid cell ARG1 expression [73]. Notably, the serum level of GM-CSF was significantly higher in mental disorder patients compared to controls [74]. It is noteworthy that the key differentially expressed cytokines also exhibit an overall negative correlation with the population of CD8^+^ T cells, suggesting that CD8^+^ T cells might be main effector responsive to SNS treatment. Actually, augmented CD8^+^ T cell activity may contribute to the amelioration of depressive symptoms, which is correlated with the decreased systemic inflammation through the elimination of cancer cells [75]. Recently, chronic stress has been shown to accelerate breast cancer progression via compromised CD8^+^ T activity, which was closely associated with gut microbiota and its metabolites [13, 76]. For instance, *Lactobacillus plantarum*-derived indole-3-lactic acid was capable of enhancing the cytotoxic activity of CD8^+^ T cells through epigenetic mechanisms [77], and microbial short-chain fatty acids (SCFAs) could modulate CD8^+^ T cell to improve adaptive immunotherapy in syngeneic murine melanoma and pancreatic cancer models [78]. Meanwhile, it is interesting to determine the precise immune cell subset by using single cell sequencing technology in the near future.

While our multi-omics analyses revealed significant SNS-associated in gut microbiota (*Lactobacillus* reduction), serum metabolites (indole decrease), and immune markers (CD8^+^ T cell increase), these findings represent a preliminary mechanistic hypothesis. The potential causal pathway—*Lactobacillus*-indole-CD8^+^ T cell axis—is worth to be explored. Preclinical studies also demonstrated that *Lactobacillus*-derived indole activates aryl hydrocarbon receptor (AhR) in immune cells to suppress CD8^+^ T cell function [60]. Meanwhile, stress-induced gut dysbiosis could elevate circulating indole levels correlating with depressive behaviors [66]. Our findings align with these mechanisms, suggesting SNS may ameliorate depression by modulating this microbial-immune crosstalk. However, future studies, such as fecal microbiota transplantation, are still required to establish the causality.

This study had some limitations. Firstly, the sample size was relatively small. Additionally, although our findings confirm that SNS improves MMD in breast cancer patients, it does not assess patients’ long-term effect of SNS. A multicenter trial with larger sample size, longer follow-up and more rigorous design is warranted. Most importantly, the omic-based correlations between *Lactobacillus* reduction, indole levels, and CD8^+^ T cell changes just represent hypothesis-generating rather than confirmatory findings. Further experimental validations are required to establish the signaling pathway.

## Conclusion

In summary, this study underscores the efficacy and safety of SNS for breast cancer patients with MMD, and highlights the potential pathway of *Lactobacillus*-Indole-CD8^+^ T cell in mediating the pharmacological activities of SNS. However, large scale clinical trials are warranted to support the application of SNS in treating breast cancer patients with MMD, and in-depth investigation is needed to understand the potential molecular mechanisms of *Lactobacillus*-Indole-CD8^+^ T signaling in mediating breast cancer progression.

## Supplementary Information

Supplementary Material 1.

## Data Availability

All the data are available by directly contacting the corresponding author, except the data provided in the article or Supplementary Material.

## Abbreviations

SNS: Si–Ni–San
MMD: Mild to Moderate Depression
TCM: Traditional Chinese medicine
USA: United States of America
HAMD-24: Hamilton Depression Scale-24
FACT-B: Functional Assessment of Cancer Therapy—Breast
PWB: Physical well-being
SWB: Social well-being
EWB: Emotional well-being
FWB: Functional well-being
AC: Additional concerns
TCMSS: Traditional Chinese medicine syndrome scale
ICD-10: International Classification of Diseases (10th version)
LQDP: Liver qi depression pattern
LFT: Liver function tests
MSE: Mental status examination
PCoA: Principal Co-ordinates Analysis
LEfSe: Linear discriminant analysis effect size
LDA: Linear discriminant analysis
PLS-DA: Partial Least Squares Discriminant Analysis
DMOA: Deep MetOrigin Analysis
