# Supplementary material for: Clinical efficacy and multi-omics analysis of Si–Ni–San for depression treatment in breast cancer patients: a randomized, double-blind, placebo-controlled, crossover trial
Source: Chin Med. 2026 Jan 6;21:9. doi: 10.1186/s13020-025-01283-y (PMC12771921; doi:10.1186/s13020-025-01283-y)
Supplement: Supplementary file 1 — Supplementary Material 1. [file 13020_2025_1283_MOESM1_ESM.docx]

**Supplementary Material**

1. **Expanded Methods**
   1. Approval letter of questionnaires
   2. CONSORT 2010 checklist
   3. Procedures and study plan
   4. Safety monitoring
   5. Patient-Completed Questionnaires
2. **Expanded Results**

sFig. 1. Data pre-processing statistics and quality control of 16S rRNA sequencing

sFig. 2. QC sample correlation analysis of metabolomic analysis

sFig. 3. Alpha diversity of patients in group A and B before and after SNS intervention

sTable 1. Safety measures after 4-week treatment with SNS or Placebo

sTable 2. Number of Patients with a given type and grade of adverse event

1. **Expanded Methods**

**1.1 Approval letter**

**
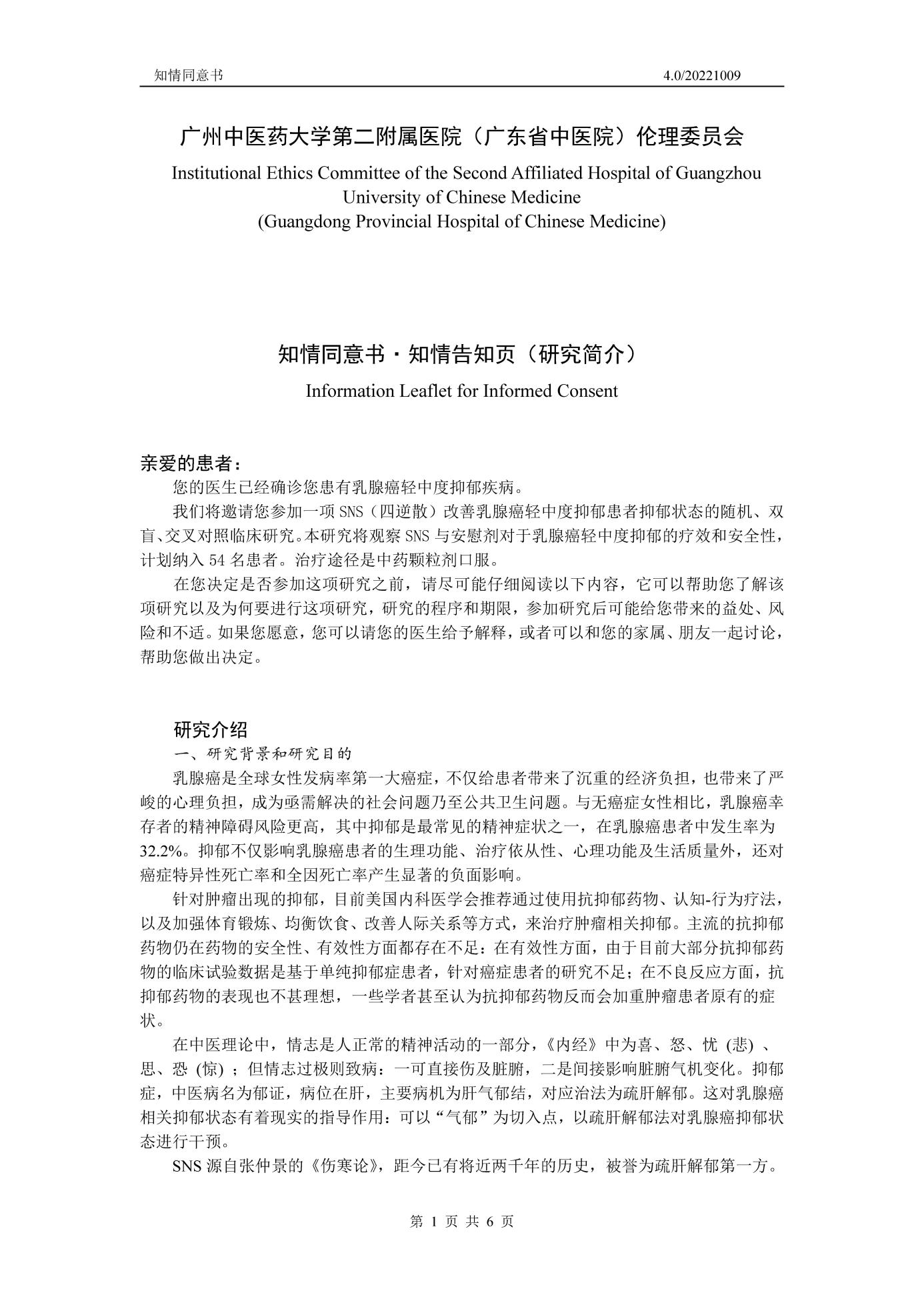
**

**1.2 CONSORT 2010 checklis**t

| **Section/Topic** | **Item No** | **Checklist item** | **Reported on page No** |
| --- | --- | --- | --- |
| **Title and abstract** | 1a | Identification as a randomised trial in the title | 1 |
|  | 1b | Structured summary of trial design, methods, results, and conclusions _(for_ _specific_ _guidance_ _see_ _CONSORT_ _for_ _abstracts)_ | 2-3 |
| **Introduction**  Background and | 2a | Scientific background and explanation of rationale | 4-7 |
| objectives | 2b | Specific objectives or hypotheses | 7 |
| **Methods**  Trial design | 3a | Description of trial design (such as parallel, factorial) including allocation ratio | 7-8 |
|  | 3b | Important changes to methods after trial commencement (such as eligibility criteria), with reasons | n/a |
| Participants | 4a | Eligibility criteria for participants | 8-9 |
|  | 4b | Settings and locations where the data were collected | 17-18 |
| Interventions | 5 | The interventions for each group with sufficient details to allow replication, including how and when they were | 10-11 |
|  |  | actually administered |  |
| Outcomes | 6a | Completely defined pre-specified primary and secondary outcome measures, including how and when they | 11-12 |
|  |  | were assessed |  |
|  | 6b | Any changes to trial outcomes after the trial commenced, with reasons | n/a |
| Sample size | 7a | How sample size was determined | 12-13 |
|  | 7b | When applicable, explanation of any interim analyses and stopping guidelines | n/a |
| Randomisation: |  |  |  |
| Sequence | 8a | Method used to generate the random allocation sequence | 9-10 |
| generation | 8b | Type of randomisation; details of any restriction (such as blocking and block size) | 9-10 |
| Allocation | 9 | Mechanism used to implement the random allocation sequence (such as sequentially numbered containers), | 9-10 |
| concealment |  | describing any steps taken to conceal the sequence until interventions were assigned |  |
| mechanism |  |  |  |
| Implementation | 10 | Who generated the random allocation sequence, who enrolled participants, and who assigned participants to | 9-10 |
|  |  | interventions |  |
| Blinding | 11a | If done, who was blinded after assignment to interventions (for example, participants, care providers, those | 9-10 |

|  |  | assessing outcomes) and how |  |
| --- | --- | --- | --- |
|  | 11b | If relevant, description of the similarity of interventions | n/a |
| Statistical methods | 12a | Statistical methods used to compare groups for primary and secondary outcomes | 17-18 |
|  | 12b | Methods for additional analyses, such as subgroup analyses and adjusted analyses | n/a |
| **Results**  Participant flow (a | 13a | For each group, the numbers of participants who were randomly assigned, received intended treatment, and | Fig. 2 |
| diagram is strongly |  | were analysed for the primary outcome |  |
| recommended) | 13b | For each group, losses and exclusions after randomisation, together with reasons | Fig. 2 |
| Recruitment | 14a | Dates defining the periods of recruitment and follow-up | 16 |
|  | 14b | Why the trial ended or was stopped | n/a |
| Baseline data | 15 | A table showing baseline demographic and clinical characteristics for each group | Table 2 |
| Numbers analysed | 16 | For each group, number of participants (denominator) included in each analysis and whether the analysis was | Fig. 2 |
|  |  | by original assigned groups |  |
| Outcomes and | 17a | For each primary and secondary outcome, results for each group, and the estimated effect size and its | Fig. 3-5, Table 3-5 |
| estimation |  | precision (such as 95% confidence interval) |  |
|  | 17b | For binary outcomes, presentation of both absolute and relative effect sizes is recommended | n/a |
| Ancillary analyses | 18 | Results of any other analyses performed, including subgroup analyses and adjusted analyses, distinguishing | n/a |
|  |  | pre-specified from exploratory |  |
| Harms | 19 | All important harms or unintended effects in each group _(for_ _specific_ _guidance_ _see_ _CONSORT_ _for_ _harms)_ | n/a |
| **Discussion**  Limitations | 20 | Trial limitations, addressing sources of potential bias, imprecision, and, if relevant, multiplicity of analyses | 32 |
| Generalisability | 21 | Generalisability (external validity, applicability) of the trial findings | 25-33 |
| Interpretation | 22 | Interpretation consistent with results, balancing benefits and harms, and considering other relevant evidence | 25-33 |
| **Other information**  Registration | 23 | Registration number and name of trial registry | 8 |
| Protocol | 24 | Where the full trial protocol can be accessed, if available | n/a |
| Funding | 25 | Sources of funding and other support (such as supply of drugs), role of funders | 31 |

*We strongly recommend reading this statement in conjunction with the CONSORT 2010 Explanation and Elaboration for important clarifications on all the items. If relevant, we also recommend reading CONSORT extensions for cluster randomised trials, non-inferiority and equivalence trials, non-pharmacological treatments, herbal interventions, and pragmatic trials. Additional extensions are forthcoming: for those and for up to date references relevant to this checklist, see [www.consort-statement.org.](http://www.consort-statement.org/)

**1.3 Procedures and study plan**

1.3.1 Initial visit

Outpatients of the Department of Mammary Disease and Psychiatry in Guangdong Provincial Hospital of Chinese Medicine will be recruited via their attending doctor and through flyers posted in the waiting room of the clinic's outpatient department. The study will include outpatients who fully meet the inclusion criteria, assessed by their attending TCM doctor and psychiatrist during a consultation. After providing informed consent, participants completed the baseline survey including cancer-related, sociodemographic questionnaires.

1.3.2 Follow-up visit

After initial visit and first assessment, patients will be called to participate in clinical visit at week 4, week 6 and week 10, respectively. At the 4^th^ and 10^th^ week visits, the patients were requested to bring back the drug bags, and the number of returned and dispensed drug bags was counted. Drug compliance was assessed by the “pill count” method, and calculated by the drug number they actually take divided by the number they are supposed to take. And patient diary recording daily intake was also provided.

1.3.3 Telephone contact

In addition to the regular follow-up visits, the patients will be followed up by telephone by the researcher at 2-week intervals to monitor the patients' psychological status, in order to detect any exacerbation of the depressive state and provide standardized clinical interventions if necessary.

1.3.4 Criteria for removal from protocol intervention

For patients whose depression worsens in the mental status examination and requires clinical intervention, they will be removed from protocol intervention and receive psychiatric treatment. The original data of all discontinued studies including the time of the last visit, the diagnosis and treatment of the psychological specialty will be recorded on the CRF form.

1.3.5 Discontinuation of treatment

For patients who withdrew from the trial in the middle of the trial, the reason for withdrawal, the treatment history and the time of the last treatment will be clearly recorded in the CRF form. For patients who miss the visit or did not come to the hospital for follow-up on time, efforts should be made to ascertain the reasons via telephone or written communication. In addition, the treatment history will be investigated and the date of the last treatment will be recorded on the CRF. The original data of all discontinued studies and dislodged cases will be kept; if dislodged due to treatment-related adverse events, the time of occurrence, treatment and final outcome need to be recorded in detail.

**1.4 Safety monitoring**

1.4.1 Liver function tests (LFT)

To prevent pharmacological liver injury, each patient will undergo liver function tests before and after 4-week SNS period and 4-week Placebo period (baseline, week 4, week 6, and week 10). In addition, any occurrence of adverse events and serious adverse events will be recorded.

1.4.2 Mental status examination (MSE)

To reduce the risk of exacerbation of depression, each patient will undergo mental status examination before and after 4-week SNS period and 4-week Placebo period (baseline, week 4, week 6, and week 10). The standardized and timely clinical interventions will be given if necessary. The 2-week telephone follow-up contact further enhances intervention safety.

1.4.3 CTCAE and Adverse event reporting

This study utilized the CTCAE (NCI Common Terminology Criteria for Adverse Events) Version 4.0 for toxicity and Reportable Adverse Event reporting. A copy of the CTCAE Version 4.0 can be downloaded from the CTEP home page (http://ctep.cancer.gov). All appropriate treatment areas should have access to a copy of the CTCAE Version 4.0.

During the study, the patients will be asked to report changes in medication and potential adverse events at every appointment (both follow-up visit and telephone contact). Adverse events were graded for severity and classified as treatment, or non-treatment related. If any abnormalities or problems, including possible adverse events (AE) or reportable adverse events, are detected during screening, subsequent measurements, or study activities, the patient will be provided appropriate care. The investigators will be responsible for recording and reporting any AEs and reportable adverse events.

In this study, we referred to a report of 150 ADR reports of Chinese patent medicines reported in the Hospital of Traditional Chinese Medicine in Chaoyang District, Beijing, China, from 2015 to 2020, focusing on the clinical manifestations of high-frequency AEs in this report.

**1.5 Patient-Completed Questionnaires**

1.5.1 HAMD-24

**Hamillton Depression Scale (HAMD-24)**

Over the last 7 days, how often have you been bothered by any of the following problems? (Use “✔” to indicate your answer)

| **Symptoms** | | None | Mild | Moderate | Severe | Very severe |
| --- | --- | --- | --- | --- | --- | --- |
| **1** | Depressed mood |  |  |  |  |  |
| **2** | Low self-esteem, guilt |  |  |  |  |  |
| **3** | Suicidal thoughts |  |  |  |  |  |
| **4** | Insomnia: initial |  |  |  |  |  |
| **5** | Insomnia: middle |  |  |  |  |  |
| **6** | Insomnia: late |  |  |  |  |  |
| **7** | Work and interests |  |  |  |  |  |
| **8** | Psychomotor retardation |  |  |  |  |  |
| **9** | Psychomotor agitation |  |  |  |  |  |
| **10** | Anxiety, psychic |  |  |  |  |  |
| **11** | Anxiety, somatic |  |  |  |  |  |
| **12** | Gastrointestinal symptoms (appetite) |  |  |  |  |  |
| **13** | Somatic symptoms, genera |  |  |  |  |  |
| **14** | Sexual disturbances |  |  |  |  |  |
| **15** | Hypochondriasis (somatisation) |  |  |  |  |  |
| **16** | Insight |  |  |  |  |  |
| **17** | Weight loss |  |  |  |  |  |
| **18** | Diurnal variation |  |  |  |  |  |
| **19** | Depersonalization and derealisation |  |  |  |  |  |
| **20** | Paranoid symptoms |  |  |  |  |  |
| **21** | Obsessional and compulsive symptoms |  |  |  |  |  |
| **22** | Helplessness |  |  |  |  |  |
| **23** | Hopelessness |  |  |  |  |  |
| **24** | Worthlessness |  |  |  |  |  |
| **Total score** | |  |  | | | |

**Hamilton Depression Scale (HAMD- 24) Manual**

| 18. Diurnal variation  0: None.  1: Mild.  2: Severe.  19. Depersonalization and derealoization  Such as: feelings of unreality, nihilistic ideas .  0: Absent.  1: Mild.  2: Moderate.  3: Severe.  4: Incapacitating.  20. Paranoid symptoms  0: None.  1: Suspicious.  2: Ideas of reference.  3: Delusions of reference and persecution.  4: Hallucinations.  21. Obsessional and compulsive symptoms  0: Absent.  1: Mild.  2: Severe. | 22. Helplessness  0: Not present.  1: Patient reports mild feelings of helplessness.  2: Moderate feelings of helplessness.  3: Strong feeling of helplessness.  4: Strong feelings of helplessness AND has given up routine activities of normal life (decreased personal hygiene, doesn’t get out of bed, difficulty feeding self, etc.)  23. Hopelessness  Pessimistic about future  0: Not present.  1: Very mild feelings of hopelessness.  2: Feels “hopeless” but accepts reassurances.  3: Expresses feelings of discouragement, despair, pessimism about future, which cannot be dispelled.  4: Inappropriately perseverates, “I’ll never get well” or equivalent.  24. Worthlessness  Ranges from mild loss of esteem, feelings of inferiority, self-deprecation to delusional notions of worthlessness.  0: Not present.  1: Very mild feelings of low self-esteem.  2: Feelings of worthlessness.  3: Strong feelings of worthlessness.  4: Delusions of worthlessness, “I am a sinner” |
| --- | --- |

**SCORING**

For 1-17, 19-20, 22-24, each question is scored on a scale of 0-5 (none, mild, moderate, severe,very severe). For 18 and 21, each question is scored on a scale of 0-2 (absent, mild, severe).

**Subscale**

**Anxiety**

Score range: 0-30, 10+11+12+13+15+17

**Weight loss**

Score range: 0-5, 16

**Cognitive disorder**

Score range: 0-27, 2+3+9+19+20+21

**Diurnal variation**

Score range: 0-20, 1+7+8+14

**Retardation**

Score range: 0-2, 18

**Sleep disorder**

Score range: 0-15, 4+5+6

**Helplessness**

Score range: 0-15, 22+23+24

**HAMD Total**

Score range: 0-114, Anxiety + Weight loss + Cognitive disorder + Diurnal variation + Retardation + Sleep disorder + Helplessness.

20-35, mild and moderate depression; ＞ 35, severe depression. The lower the score, the milder depressive symptoms.

Reference: Hamilton M. Rating depressive patients. J Clin Psychiatr. 1980;41(12 Pt2):21–4

1.5.2 FACT-B

**Functional Assessment of Cancer Therapy–Breast (FACT-B)**

Below is a list of statements that other people with your illness have said are important. Use “✔” to indicate your response as it applies to the past 7 days.

|  | | Not at all | A little bit | Some-what | Quite a bit | Very much |
| --- | --- | --- | --- | --- | --- | --- |
| **PHYSICAL WELL-BEING** | | | | | | |
| GP1 | I have a lack of energy |  |  |  |  |  |
| GP2 | I have nausea |  |  |  |  |  |
| GP3 | Because of my physical condition, I have trouble meeting the needs of my family |  |  |  |  |  |
| GP4 | I have pain |  |  |  |  |  |
| GP5 | I am bothered by side effects of treatment |  |  |  |  |  |
| GP6 | I feel ill |  |  |  |  |  |
| GP7 | I am forced to spend time in bed |  |  |  |  |  |
| **SOCIAL/FAMILY WELL-BEING** | | | | | | |
| GS1 | I feel close to my friends |  |  |  |  |  |
| GS2 | I get emotional support from my family |  |  |  |  |  |
| GS3 | I get support from my friends |  |  |  |  |  |
| GS4 | My family has accepted my illness |  |  |  |  |  |
| GS5 | I am satisfied with family communication about my illness |  |  |  |  |  |
| GS6 | I feel close to my partner (or the person who is my main support) |  |  |  |  |  |
| QL | *Regardless of your current level of sexual activity, please answer the following question. If you prefer not to answer it, please mark this box □ and go to the next section.* |  |  |  |  |  |
| GS7 | I am satisfied with my sex life |  |  |  |  |  |
| **EMOTIONAL WELL-BEING** | | | | | | |
| GE1 | I feel sad |  |  |  |  |  |
| GE2 | I am satisfied with how I am coping with my illness |  |  |  |  |  |
| GE3 | I am losing hope in the fight against my illness |  |  |  |  |  |
| GE4 | I feel nervous |  |  |  |  |  |
| GE5 | I worry about dying |  |  |  |  |  |
| GE6 | I worry that my condition will get worse |  |  |  |  |  |
| **FUNCTIONAL WELL-BEING** | | | | | | |
| GF1 | I am able to work (include work at home) |  |  |  |  |  |
| GF2 | My work (include work at home) is fulfilling |  |  |  |  |  |
| GF3 | I am able to enjoy life |  |  |  |  |  |
| GF4 | I have accepted my illness |  |  |  |  |  |
| GF5 | I am sleeping well |  |  |  |  |  |
| GF6 | I am enjoying the things I usually do for fun |  |  |  |  |  |
| GF7 | I am content with the quality of my life right now |  |  |  |  |  |
| **ADDITIONAL CONCERNS** | | | | | | |
| B1 | I have been short of breath |  |  |  |  |  |
| B2 | I am self-conscious about the way I dress |  |  |  |  |  |
| B3 | One or both of my arms are swollen or tender |  |  |  |  |  |
| B4 | I feel sexually attractive |  |  |  |  |  |
| B5 | I am bothered by hair loss |  |  |  |  |  |
| B6 | I worry that other members of my family might someday get the same illness I have |  |  |  |  |  |
| B7 | I worry about the effect of stress on my illness |  |  |  |  |  |
| B8 | I am bothered by a change in weight |  |  |  |  |  |
| B9 | I am able to feel like a woman |  |  |  |  |  |

**SCORING**

Each question is scored on a scale of 0-4 (not at all, a little bit, somewhat, quite a bit, very much). All items are divided into 2 categories: forward and reverse, the score calculated differently. Among them, GP1-GP7, GE1, GE3-GE6, B1-B3, B5-B8 are reverse entries, the others are positive entries. Forward item score = (0 + Item response) reverse item score = (4 - Item response).

**Subscale**

**PHYSICAL WELL-BEING (PWB)**

Score range: 0-28, GP1+GP2+GP3+GP4+GP5+GP6+GP7

**SOCIAL/FAMILY WELL-BEING (SWB)**

Score range: 0-28, GS1+GS2+GS3+GS4+GS5+GS6+GS7

**EMOTIONAL WELL-BEING (EWB)**

Score range: 0-24, GE1+GE2+GE3+GE4+GE5+GE6

**FUNCTIONAL WELL-BEING (FWB)**

Score range: 0-28, GF1+GF2+GF3+GF4+GF5+GF6+GF7

**ADDITIONAL CONCERNS (AC)**

Score range: 0-36, B1+B2+B3+B4+B5+B6+B7+B8+B9

**FACT-B Total**

Score range: 0-144, PWB+SWB+EWB+FWB+AC. The higher the score, the better the QOL.

Reference: Brady MJ, et al. Reliability and validity of the Functional Assessment of Cancer Therapy-Breast quality-of-life instrument. J Clin Oncol. 1997 Mar;15(3):974-86.

1.5.3 TCMSS

**Traditional Chinese Medicine Syndrome Score Scale (TCMSSS)**

Over the last 2 weeks, how often have you been bothered by any of the following problems? (Use “✔” to indicate your answer)

| **Symptoms** | | A little of the time | Some of the time | Good part of the time | Most of the time |
| --- | --- | --- | --- | --- | --- |
| ***Primary symptom*** | | | | | |
| **P1** | Depressive symptoms |  |  |  |  |
| **P2** | Oppression in the chest |  |  |  |  |
| **P3** | Frequent sighing |  |  |  |  |
| **P4** | Fullness and discomfort in the chest and rib-side |  |  |  |  |
| **P5** | Distending pain in the breasts |  |  |  |  |
| ***Secondary symptom*** | | | | | |
| **S1** | Gastric stuffiness or abdominal pain and borborigmus |  |  |  |  |
| **S2** | Frequent belching |  |  |  |  |
| **S3** | Impatience and irascibility |  |  |  |  |
| **S4** | Headache and eye pain |  |  |  |  |
| **S5** | Bitter taste in the mouth |  |  |  |  |
| **S6** | Hard stool and dark urine |  |  |  |  |
| **S7** | Mind-wandering state |  |  |  |  |
| **S8** | Profuse dreaming and light sleep |  |  |  |  |
| **S9** | Sorrow, anxiety and fragile |  |  |  |  |
| **S10** | Symptom fluctuates with mood |  |  |  |  |
| **Tongue and pulse manifestation** | | present | | not present | |
| **TP1** | Light white fur |  | |  | |
| **TP2** | String-like pulse |  | |  | |
| **Total score** | |  | | | |

**SCORING**

For P1-P5 and S1-S10, each question is scored on a scale of 0, 2, 4, 6 (a little of the time, some of the time, good part of the time, most of the time). For TP1-TP2 and S1-S10, each question is scored on a scale of 0, 2 (present, not present).

**Subscale**

**Primary symptom**

Score range: 0-30, P1+P2+P3+P4+P5

**Secondary symptom**

Score range: 0-60, S1+S2+S3+S4+S5+S6+S7+S8+S9+S10

**Tongue and pulse manifestation**

Score range: 0-4, TP1+TP2

**TCMSS Total**

Score range: 0-94, Primary symptom + Secondary symptom + Tongue and pulse manifestation. The lower the score, the milder clinical symptoms.

Reference: The Chinese National Administration of Traditional Chinese medicine. TCM Diagnostic and therapeutic criteria for disease and pattern[M]. NANJING UNIVERSITY PRESS,1994.

The Chinese National Medical Products Administration. The guiding principles for clinical research on TCM(GGTG-2015-12187). 2015. Link: https://www.nmpa.gov.cn/xxgk/ggtg/ypggtg/ypqtggtg/20151103120001444.html

1. **Expanded Result**


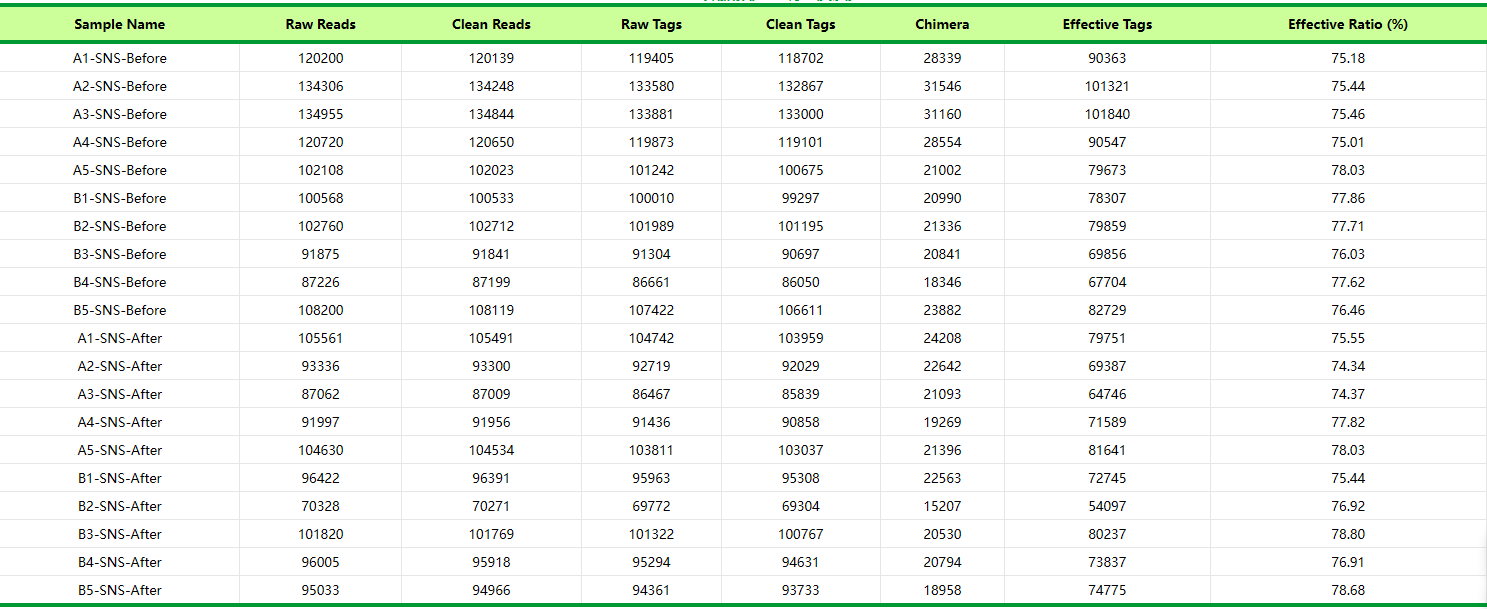


**sFig. 1.Data pre-processing statistics and quality control of** ***16S rRNA sequencing.*** Generally, the amount of effective tags is more than 30,000 reads to meet the needs of sequencing

**
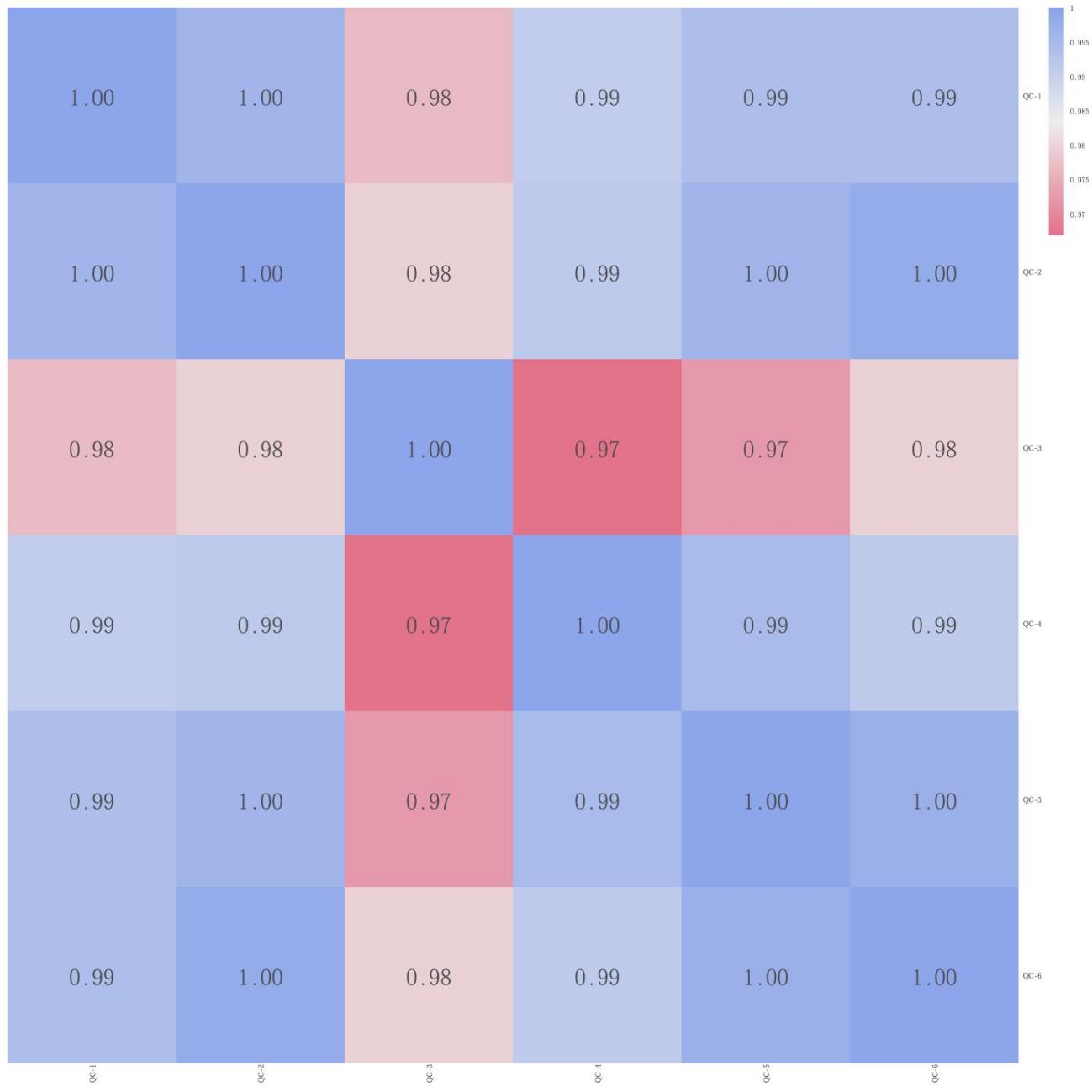
**

**sFig. 2. QC sample correlation analysis of m*etabolomic analysis.*** Pearson correlation analysis was performed on QC samples. The higher the correlation of QC samples (the closer the R is to 1) , the better stability of the whole testing process and the higher quality of data.


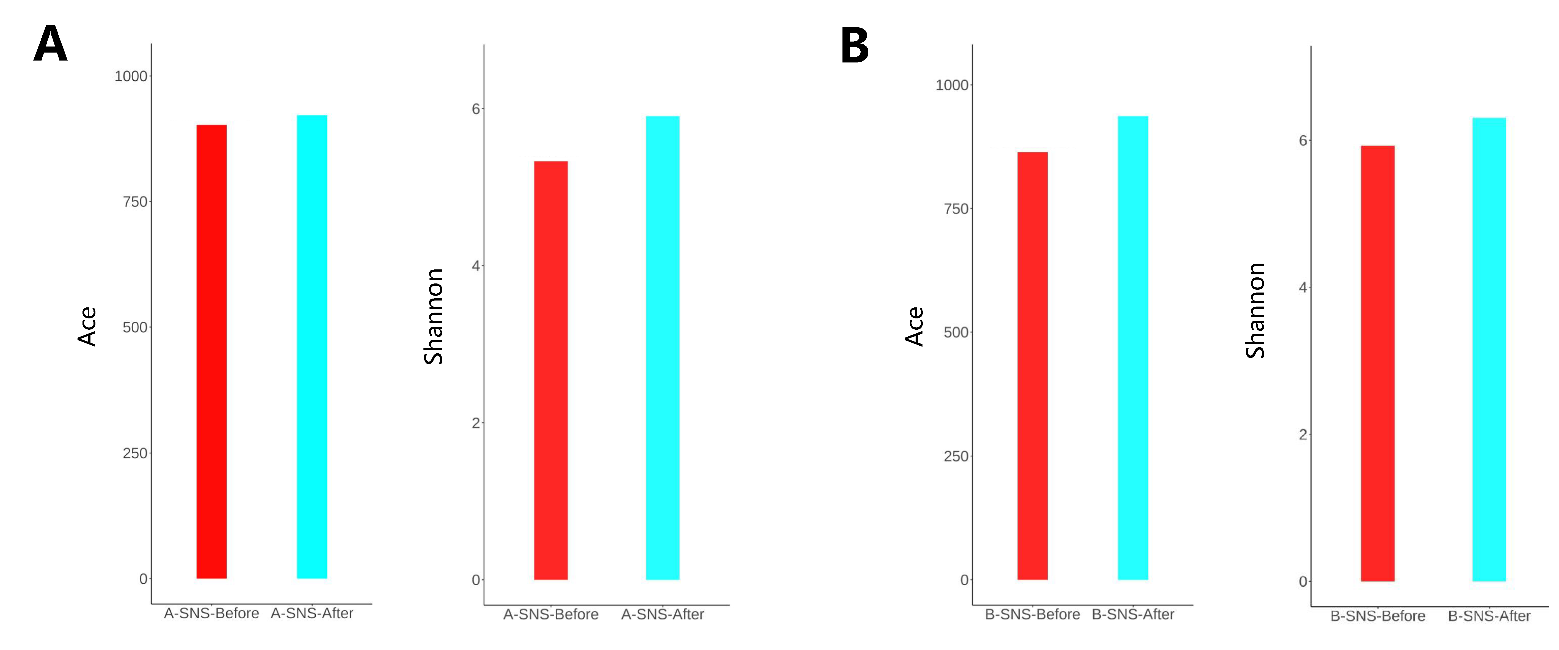


**sFig. 3. Influence of SNS on Alpha diversity of breast cancer patients with MMD.**  (A) Changes of Ace and Shannon index before and after SNS intervention in Group A (n=5); (B) Changes of Ace and Shannon index before and after SNS intervention in Group B (n=5).

**sTable 1. Safety measurement after four-week treatment with SNS or placebo**

| **Safety measures** | **SNS (n=53)** | **Placebo (n=53)** |
| --- | --- | --- |
| **Liver function（ALT, AST）** |  |  |
| **Normal** | **53 (100%)** | **53 (100%)** |
| **Abnormal** | **0 (0%)** | **0 (0%)** |
| **Mental status examination** |  |  |
| **Normal** | **53 (100%)** | **53 (100%)** |
| **Exacerbation** | **0 (0%)** | **0 (0%)** |

**sTable 2. Number of patients with a given type and grade of adverse event**

| **Adverse event** | **SNS (n=53)** | | | | | | **Placebo (n=53)** | | | | | |
| --- | --- | --- | --- | --- | --- | --- | --- | --- | --- | --- | --- | --- |
|  | 0 | 1 | 2 | 3 | 4 | 5 | 0 | 1 | 2 | 3 | 4 | 5 |
| **Erythra** | 53 | 0 | 0 | 0 | 0 | 0 | **53** | 0 | 0 | 0 | 0 | 0 |
| **Pruritus** | 53 | 0 | 0 | 0 | 0 | 0 | **53** | 0 | 0 | 0 | 0 | 0 |
| **Abdominal pain** | 53 | 0 | 0 | 0 | 0 | 0 | **53** | 0 | 0 | 0 | 0 | 0 |
| **Diarrhea** | 52 | 1 | 0 | 0 | 0 | 0 | **53** | 0 | 0 | 0 | 0 | 0 |
| **Constipation** | 53 | 0 | 0 | 0 | 0 | 0 | **53** | 0 | 0 | 0 | 0 | 0 |
| **Decreased appetite** | 53 | 0 | 0 | 0 | 0 | 0 | **53** | 0 | 0 | 0 | 0 | 0 |
| **Nausea** | 53 | 0 | 0 | 0 | 0 | 0 | **53** | 0 | 0 | 0 | 0 | 0 |
| **Somnolence** | 53 | 0 | 0 | 0 | 0 | 0 | **53** | 0 | 0 | 0 | 0 | 0 |
| **Insomnia** | 53 | 0 | 0 | 0 | 0 | 0 | **53** | 0 | 0 | 0 | 0 | 0 |
| **Dizziness** | 53 | 0 | 0 | 0 | 0 | 0 | **53** | 0 | 0 | 0 | 0 | 0 |
| **Headache** | **53** | 0 | 0 | 0 | 0 | 0 | **53** | 0 | 0 | 0 | 0 | 0 |
| **Chest distress** | 53 | 0 | 0 | 0 | 0 | 0 | **53** | 0 | 0 | 0 | 0 | 0 |
| **Asthma** | 53 | 0 | 0 | 0 | 0 | 0 | **53** | 0 | 0 | 0 | 0 | 0 |
| **Palpitation** | **53** | 0 | 0 | 0 | 0 | 0 | **53** | 0 | 0 | 0 | 0 | 0 |
| **Agitation** | **53** | 0 | 0 | 0 | 0 | 0 | **53** | 0 | 0 | 0 | 0 | 0 |
| **Dry mouth** | **53** | 0 | 0 | 0 | 0 | 0 | 52 | 1 | 0 | 0 | 0 | 0 |
| **Transient face** | **53** | 0 | 0 | 0 | 0 | 0 | **53** | 0 | 0 | 0 | 0 | 0 |
| **Fever** | **53** | 0 | 0 | 0 | 0 | 0 | **53** | 0 | 0 | 0 | 0 | 0 |
